# Tumor-Derived Exosomes Enriched by miRNA-124 Promote Anti-tumor Immune Response in CT-26 Tumor-Bearing Mice

**DOI:** 10.3389/fmed.2021.619939

**Published:** 2021-04-27

**Authors:** Ramazan Rezaei, Kaveh Baghaei, Seyed Mahmoud Hashemi, Mohammad Reza Zali, Hossein Ghanbarian, Davar Amani

**Affiliations:** ^1^Department of Immunology, School of Medicine, Shahid Beheshti University of Medical Sciences, Tehran, Iran; ^2^Gastroenterology and Liver Diseases Research Center, Research Institute for Gastroenterology and Liver Diseases, Shahid Beheshti University of Medical Sciences, Tehran, Iran; ^3^Department of Tissue Engineering and Applied Cell Sciences, School of Advanced Technologies in Medicine, Shahid Beheshti University of Medical Sciences, Tehran, Iran; ^4^Department of Medical Biotechnology, School of Advanced Technologies in Medicine, Shahid Beheshti University of Medical Sciences, Tehran, Iran

**Keywords:** tumor-derived exosomes, MiR-124-3p, anti-tumor immune response, immunotherapy, colon cancer, CT-26

## Abstract

Exosomes have been introduced as a new alternative delivery system for the transmission of small molecules. Tumor-derived exosomes (TEXs) not only contain tumor-associated antigens to stimulate antitumor immune responses but also act as natural carriers of microRNAs. The aim of the current study was to evaluate the efficacy of miR-124-3p-enriched TEX (TEXomiR) as cell-free vaccine in the induction of antitumor immune responses in a mouse model of colorectal cancer. Briefly, the exosomes were isolated from cultured CT-26 cell line, and modified calcium chloride method was used to deliver miR-124-3p mimic into the exosomes. We used a CT-26-induced BALB/c mouse model of colorectal cancer and analyzed the effect of TEXomiR on survival, tumor size, spleen and tumor-infiltrated lymphocytes, and splenocyte proliferation. Furthermore, intra-tumor regulatory T cells, cytotoxic activity of the splenocytes, and cytokine secretion was also evaluated to describe the anti-tumor immune response. When the tumor size reached 100 mm^3^, the mice were injected with TEXomiR, TEX, and/or phosphate-buffered saline (PBS) subcutaneously three times with 3-day interval, and then tumor size was monitored every 2 days. The *in vitro* results indicated that TEXs could efficiently deliver functional miR-124-3p mimic. The *in vivo* evaluation in tumor-bearing mice showed that treatment with TEXomiR can elicit a stronger anti-tumor immune response than unloaded TEX and PBS. Significant tumor growth inhibition and increased median survival time was achieved in tumor-bearing mice treated with TEXomiR. A significant decrease in CD4/CD8 and Treg/CD8 ratio in tumor tissue was demonstrated. Moreover, increased cytotoxicity and proliferation of splenocytes in the TEXomiR group compared to the TEX and PBS groups were identified. Taken together, our data demonstrated that tumor-derived exosomes efficiently deliver miR-124-3p mimic, and TEXomiR promotes anti-tumor immune responses.

## Introduction

Colorectal cancer (CRC) is the third leading cause of deaths among all human cancers. Its overall incidence is ~5%, and the 5-year survival rate varies from 40 to 60% (1). Consequently, it represents a critical health burden. Most investigations on CRC have focused on genetic and epigenetic alterations in protein-coding genes for their important function in CRC etiopathogenesis (2, 3). However, microRNAs as a class of small non-coding RNAs have attracted much attention (4).

Changes in the expression profile of miRNAs in CRC have been well-documented (5). Several studies have shown that miRNAs can act as tumor suppressors or oncogenes and are commonly dysregulated in tumor (5, 6). miRNAs play an essential role in several fundamental cellular processes, including proliferation, differentiation, apoptosis, and invasion (7, 8). Several studies have evaluated microRNA expression profiles in CRC and authenticated that microRNAs are reproducibly and consistently altered in this malignancy (9, 10). Up to now, dysregulation of the 164 microRNAs has been illustrated in CRC; ~66% of them were increased, and the remaining were decreased in this disease (5).

From tumor-suppressive microRNA, miR-124 has attracted much attention, and a number of studies in the recent years focus on the interaction of this molecule with various transcription factors and its important role in CRC (11, 12). MiR-124 through various cascade pathways, including the PTB1/PKM1/PKM2 pathway (13), or by targeting the STAT-3 molecule inhibits CRC tumor growth (12). Furthermore, miR-124, by targeting STAT-3 (12) and SOCS5 (14), promotes T cell-dependent immune response in tumor (15, 16). Indeed miR-124 increases the differentiation of CD4+ T cells into Th1 and Th17 by targeting the SOCS-5 transcription factor and increases the expression of cytokines IL-2, IFN-γ, and TNF-α (14). Decreased expression of miR-124 in several cancers including CRC has been reported (17). This decrease affects the expression of the post-transcriptional regulation of target mRNAs (such as E-cadherin, P16), which eventually leads to colonic tumorigenesis and its progression (18). One of the main obstacles today in the development of clinical application of miRNA in various diseases is the lack of an effective and efficient delivery system (19).

In the last decade, exosomes have been presented as a new alternative delivery system for the transmission of microRNAs and siRNAs as therapeutic molecules (19). Exosomes are natural nanovesicles with an approximate size of 30–150 nm and which have unique characteristics such as low toxicity, nano-scale size, immune compatibility, homogenous, and relatively stable (20). Exosomes are secreted by all cell types through inward budding of the inner endosomal membrane followed by plasma membrane fusion (20). Clinically, tumor cells are capable of secreting miRNA-containing exosomes that are taken up by the recipient or target cell and alter its function (21). Exosomes which are derived from tumor cells not only contain tumor-specific and tumor-associated antigens to stimulate antitumor immune responses but also act as natural carriers of microRNA. Exosomes can be used for immunotherapy by altering their genetic contents (21, 22). Given the high incidence and mortality rate of colorectal cancer and the low efficacy of current treatments, the aim of this study was to evaluate the efficacy of miR-124-3p-enriched tumor exosomes (TEXomiR) as a cell-free vaccine to enhance antitumor immune responses in a CT-26-induced mouse model of colorectal cancer.

## Materials and Methods

### Cell Line

CT-26 cells, as a murine colorectal carcinoma cell line, were purchased from the Pasteur Institute of Iran. CT26 is an adherent, fibroblast-like-morphology cell with high tumorigenicity and invasiveness (23). The cells were cultured in complete RPMI1640 media supplemented with 10% fetal bovine serum, 1% penicillin–streptomycin (10,000 U/ml), and 1% L-glutamine. The CT-26 cells were maintained under a humidified atmosphere of 5% CO_2_ in air at 37°C. After that, the cells were passaged by trypsinization when 80% confluency was reached at seeding densities of 4 × 10^5^ cells/ml.

### Mice

Six- to 8-week-old female BALB/c mice were provided from the Pasteur Institute (Karaj, Iran). The animals were raised, treated, and handled according to the ethical protocols of animal treating and handling of the Institutional Ethical Committee and Research Advisory Committee of Shahid Beheshti University of Medical Sciences (IR.SBMU.MSP.REC.1397.741). The mice were maintained in cages held at room temperature (22–24°C) in a ventilated room and with unrestricted access to water and food.

### Cell Adaptation and Isolation of Exosomes

Due to the presence of exosomes in fetal bovine serum (FBS), for resolving their interference with tumor exosomes, the CT-26 cells were adapted to FBS-free medium through sequential adaptation. The adaptation procedure was started with cells in the exponential growth phase at a cell density of 1.25 × 10^5^ cells/ml and a cell viability of > 90%. The percentage of FBS was gradually reduced from 10 to 0% over 10 days in the culture medium. When the cells were completely stabled in a serum-free medium without significant morphological changes, the adapted cells were cultured in T75 flasks. The flasks were kept in the incubator at 37°C for 72 h, and then conditioned media (CM) was collected for exosome isolation. The exosomes were isolated from CM using Exocib kit (Cibzist, Tehran, Iran) according to the manufacturer's procedure and stored at −80°C. Briefly, cell debris and particles were removed by centrifuging at 300 × *g* for 10 min, and then the samples were filtered through a 0.22-μm filter following the manufacturer's instruction.

### Characterization of Isolated Exosomes

The protein concentration of exosomes was measured by bicinchoninic acid (BCA) protein quantification kit (DNAbioTech, Tehran, Iran). The average exosome yield was 1,525 μg from 150 ml (5–7 × 10^7^ cells) of culture supernatant. The morphological assessment of the isolated exosomes was evaluated using transmission electron microscopy (TEM) at 80 kV. Briefly, the isolated exosomes were resuspended in phosphate-buffered saline (PBS) and fixed for 1 h with 2% paraformaldehyde at room temperature. The fixed exosomes were loaded on TEM grids treated with UV light to resolve static electricity. For scanning electron microscopy (SEM), the exosomes were fixed with 2.7% glutaraldehyde for 15 min. After that, the exosomes were washed with PBS and then dehydrated with an ascending sequence of ethanol (40, 60, 80, and 98%). The samples were left to dry at room temperature and then analyzed by SEM. Nano-zetasizer (Malvern Corp., Malvern, UK) was applied to distinguish the zeta potential and size distribution of exosomes. The data were analyzed using Zetasizer software v7.11 (Malvern Corp, Malvern, UK).

### Loading the Exosomes With miR-124-3p Mimic

In order to load the exosomes with miR-124-3p, a modified calcium chloride method was used (24). The mixture was prepared by mixing exosomes and miR-124 mimic [200 pmol in 1:1 (wt/wt) ratio in CaCl_2_ solution (final concentration 0.1 M)]. The final concentration of exosomes in the mixture was adjusted to 0.5 μg/μl, and the final volume was set to 300 μl. The mixture was moved to ice for 30 min and then heat-shocked at 42°C for 60 s. For an additional 5 min, the mixture was moved to ice. After transfection, one unit of RNase A (Thermo Fisher) was added to the mixture to eliminate free and unloaded microRNAs. In the next step, the miR-124-enriched exosomes (TEXomiR) were purified by employing the Exocib kit protocol. Finally, the encapsulation efficiency of miR-124 mimic in the loaded exosomes was conducted by real-time PCR, and the miRNA loading efficiency was calculated by the following formula.

$$\begin{array}{l} \text{miRNA loading efficiency }=\;\frac{\text{ Vc Cc MWc}}{\text{ Vs Cc MWs}}\;\times \frac{\text{REc}}{\text{REs}} \end{array}$$

*V*_c_, volume of exogenous control; *C*_c_, concentration of exogenous control; MW_c_, molecular weight of exogenous control; *V*_s_, volume of transfected miRNA; *C*_s_, concentration of transfected miRNA; MW_s_, molecular weight of transfected miRNA; RE_s_, relative expression of miRNA-124 mimic; RE_c_, relative expression of control.

### RNA Extraction, mRNA, and miRNA Analysis

Total RNA was extracted using miRNeasy_Mini RNA Extraction Kit (Qiagen, Hamburg, Germany) according to the manufacturer's procedures and stored at −80°C until use. cDNA was synthesized with miScript II RT Kit (Qiagen, Hamburg, Germany). The expression levels of miRNA-124 were determined using the SYBR Green Master Mix Kit (Ampliqon, Odense, Denmark), and the expression levels of potential miRNA-124 targets, STAT-3, SP-1, and SOCS5, were conducted by applying SYBR green Master Mix on a Rotor-Gene Q real-time PCR machine (Qiagen, Hamburg, Germany). The expression levels of STAT-3, SOCS5, and SP1 were normalized to the GAPDH gene, and miR-124 expression was normalized to U6 snRNA as an endogenous control by using 2^−ΔΔCt^ method. The gene-specific primer sets for STAT-3, SOCS5, and SP1 and GAPDH as a housekeeping gene were designed using the NCBI Primer-Blast Tool. The primer mix of U6 and miR-124 was provided from Exiqon (Vedbaek, Denmark). All results are representative of three independent experiments.

### Cell Viability Assay

Cell viability was ascertained by applying the MTT assay according to the manufacturer's protocol (Sigma-Aldrich, Irvine, UK). Before treatment, CT-26 cell lines were seeded at a concentration of 10^4^ cells/well in a 96-well plate. After overnight seeding, the cells were treated with unloaded TEX (exosomes isolated from CT-26 cell line) and TEXomiR in five different concentrations (5, 10, 15, 20, and 25 μg) for 24 and 48 h. Triplicate results were read by an ELISA reader at 570 nm (TECAN, Salzburg, Austria).

### Apoptosis Detection by Flow Cytometry

Cell apoptosis detection was conducted by FITC Annexin V Apoptosis Detection Kit with PI (BioLegend, San Diego, USA) according to the manufacturer's instruction. Briefly, CT-26 cell lines in treated groups, TEX, and TEXomiR were harvested and washed with BioLegend's cell staining buffer and were then resuspended in Annexin V binding buffer at a density of 0.5–1.0 × 10^6^ cells/ml. Next, 100 μl of cell suspension was transferred in a 5-ml test tube. Subsequently, 5 μl of FITC Annexin V and 10 μl of propidium iodide solution were added to the cells of each tube. The tubes were vortexed and incubated for 15 min at 25°C in the dark. Finally, 400 μl of Annexin V binding buffer was added to each tube and was then analyzed by a flow cytometer (FACSCalibur, BD Company, San Diego, CA, USA) and flowjo software (FlowJo, LLC, Ashland, OR, USA).

### *In vivo* Anti-tumor Immune Response Analyses

**Figure 1:**
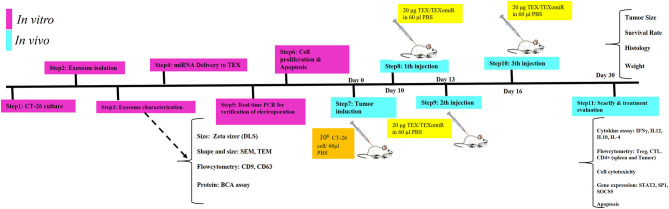
Schematic illustration of the study design. *In vitro* and *in vivo* assay for the evaluation of anti-tumor effects of miR-124-enriched tumor exosomes.

Female BALB/c mice were raised and maintained at room temperature (22–24°C) in a ventilated room with unrestricted access to water and food. For tumor induction, 1 × 10^6^ CT-26 cells were suspended in 60 μl of PBS and subcutaneously injected into the left flank region of BALB/c mice. At 1 week after CT-26 cell inoculation, when the tumors were palpable, the CT-26 tumor-bearing mice were randomly allocated into three different groups (eight mice in each group). To evaluate whether immunization with TEXomiR could inhibit tumor growth, CT-26 tumor-bearing mice were injected with TEXomiR three times with 3-day interval, and then tumor size was monitored every 2 days (Figure 1). The treatment groups included PBS, TEX, and TEXomiR. After the tumor size reached 100 mm^3^, the mice were subcutaneously injected into the right flank with 20 μg of TEX, 20 μg of TEXomiR, or with PBS as a control on days 10, 13, and 16 from CT-26 inoculation. Tumor size was monitored every 2 days using a digital caliper, and tumor volume was measured according to the following formula: length × width^2^ × 0.5. The mice were sacrificed 2 weeks after the last injection. Three mice from each group were used to evaluate the survival rate. The survival of mice was monitored for 60 days in CT-26 tumor-bearing mice. When the tumor volume reached 2,000 mm^3^, the animals were sacrificed by decapitation after a short contact with carbon dioxide, and then tumor tissues were surgically resected and weighed. Kaplan–Meier method was employed to assess the probability of mice survival.

### Flow Cytometry Analysis of Intra-tumor and Lymph Node T Cells

The mice were sacrificed, and the tumor tissues and lymph nodes were collected and fragmented into small pieces, rinsed twice with cold PBS, and minced with scalpel and forceps. The lymph node cells were passed through a 100-μm filter to achieve a single-cell suspension. After that, the erythrocytes were lysed by applying ACK lysis buffer (Na_2_EDTA, NH_4_Cl, and KHCO_3_). The remaining cells were washed with cold PBS and resuspended in RPMI 1640 medium. The prepared mixture of lymphocytes was stained by immunofluorescence staining. The monoclonal antibodies used for immunofluorescence staining were as follows: PE-conjugated anti-CD4 (BioLegend, San Diego, USA), FITC-conjugated anti-CD8 (BioLegend, San Diego, USA), PerCP-conjugated anti-CD3 (BioLegend, San Diego, USA), FITC-conjugated anti-CD25 (BioLegend, San Diego, USA), and APC-conjugated anti-Foxp3 (BioLegend, San Diego, USA). The cells were stained in a tube containing 1 × 10^5^ cells, and then the tubes were placed on ice. Briefly, 1 μl from each antibody was added to the tubes and placed in the dark for 30 min. The tubes were then centrifuged at 1,000 *g* for 5 min and rinsed with PBS, and the supernatant was discarded. The staining buffer was then added to the cells and analyzed with a flow cytometer. The isolation of T lymphocytes from mouse tumor tissues was adopted from a previous report (21). For evaluation of tumor-infiltrating lymphocytes, tumor tissues were collected and fragmented into small pieces, rinsed twice with cold PBS, gently passed through a steel mesh with a sterile syringe, and incubated with collagenase type IV (0.05 mg/ml) (Worthington Biochem, Lakewood, NJ) for 40 min at 37°C. The digested suspension was passed through a 70-μm cell strainer. The eluted cells were centrifuged at 530 *g* for 8 min, and the supernatant was discarded. The pellets were resuspended with 10 ml Percoll 40% (Pharmacia, Uppsala, Sweden) and then centrifuged at 850 *g* for 30 min. The erythrocytes in the cell pellets were lysed by applying an ACK lysis buffer, and the tumor-infiltrated lymphocytes were stained with immunofluorescence staining as described above.

### Lymphocyte Proliferation Test

After the isolation of splenocytes from sacrificed mice, splenocytes were seeded in 96-well plates, at a density of 5 × 10^5^ cells/well, in the presence of 4 μg/ml polyhydroxyalkanoate (PHA), 20 μg/ml TEX, and 20 μg/ml tumor lysate or media. Subsequently, the plates were incubated for 48 h at 37°C in 5% CO_2_. Splenocyte proliferation in different groups was measured by MTT assay. For MTT test, 20 μl MTT solution (5 mg/ml) was added to each well and incubated for 4 h. After that, 100 μl dimethyl sulfoxide was added, and the formazan crystals were dissolved by pipetting. Finally, absorbance at 570 and 630 nm (reference) was measured by ELISA reader. OD difference between test and reference was used as a survival index. In addition, the stimulation index (SI) was calculated by the following formula:

$$\begin{array}{l} Stimulation\;index\;\left(\text{SI}\right)=\;\frac{\text{OD }\left(\text{mean of treated cells}\right)\;}{\text{OD }\left(\text{mean of negative control}\right)\;} \end{array}$$

### *In vitro* Cytotoxic Activity of Splenocytes

The cytolytic activity of the splenocytes was conducted by FITC Annexin V Apoptosis Detection Kit (BioLegend, San Diego, USA). At 2 weeks after the last inoculation, splenocytes as effector cells were extracted, and a single-cell suspension was collected. CT-26 cells were used as target cells. An approximate number of 1 × 10^4^ CT-26 cells (100 μl) were co-cultured in a 96-well plate with effector cells (8 h at 37°C) in a volume of 100 μl at target/effector ratios of 1:10 and 1:20. After that, CT-26 cells were harvested and washed with BioLegend's Cell Staining Buffer, and then these were resuspended in Annexin V Binding Buffer at a density of 0.5–1.0 × 10^6^ cells/ml. Next, 100 μl of cell suspension was transferred in a 5-ml test tube. Subsequently, 5 μl of FITC Annexin V and 10 μl of propidium iodide solution were added to the cells of each tube. The tubes were vortexed and incubated for 15 min at 25°C in the dark. Finally, 400 μl of Annexin V binding buffer was added to each tube, and then this was analyzed by flow cytometry and flowjo software.

### Evaluation of Cytokine Production by Enzyme-Linked Immunosorbent Assay

In order to evaluate the secretory cytokines of spleen cells after stimulation, spleen cells in different groups were stimulated by PBS, tumor lysate, PHA (Gibco, NY, USA), and TEX. For this purpose, 1 × 10^6^ spleen cells from each mouse were cultured in 500 μl of complete RPMI1640 medium on 24-well plates for 72 h. The spleen cells of each mouse were cultured in four different wells. In the first well, the cells were treated with PBS; in the second well, the cells were treated with 20 μg tumor lysate; in the third well, the spleen cells were treated with 4 μg/ml PHA; and in the last well, the spleen cells were treated with 20 μg/ml TEX. The cells were cultured for 72 h at 37°C in a humidified 5% CO_2_ atmosphere. After that, supernatant was collected, and the amount of IL12p70, IFN-γ, IL-4, and IL10 cytokines in different groups (TEXomiR, TEX, and PBS) was measured by a specific ELISA assay kit (BioLegend, San Diego, USA) according to the manufacturer's instructions.

### Histology

The tissue samples including lymph node, lung, liver, and tumor were embedded in paraffin and cut into 5-μm-width sections. The tissue sections were deparaffinized, and the sections were stained with hematoxylin–eosin (H&E) staining for evaluation of possible metastasis in the lung, liver, and adjacent lymph nodes to the tumor. The rate of lymphocyte infiltration and tumor necrosis in the tumor tissues was evaluated. The picture of each field from different sections was taken under a light microscope, and the cell nuclei in each field were quantified by using Image J software. For grading of tumor, mitotic activity was calculated (25, 26). Mitotic counts (per 10 hpf) were evaluated in at least 10 fields in areas of highest mitotic activity (26). Tumor grade is then allocated on the following basis:

3–5 points: grade I—well differentiated
6–7 points: grade II—moderately differentiated
8–9 points: grade III—poorly differentiated

### Immunohistochemistry

Immunohistochemistry (IHC) was performed to determine the expression of CD3 and caspase 3 in tumor tissue. Tumor samples were deparaffinized in ethanol and xylene and then submerged in 10% hydrogen peroxide for 10 min. The samples were immersed in tris buffer (pH = 7.4) and subsequently incubated in citrate acid buffer (pH = 6) for 40–45 min. In the next step, blocking was conducted with 2% bovine serum albumin for 15 min and stained with CD3 and caspase 3 primary antibody at 4°C overnight. Subsequently, the samples were washed three times with tris buffer (pH = 7.4) and incubated with secondary antibody for 30 min at 25°C. After that, the tumor tissues were washed with tris buffer, and positive regions were detected with the HRP-DAB detection kit. These stained samples were visualized under a Nikon microscope (NIKON, Japan), and illustrative images were taken with a digital camera. Finally, the mean intensity of CD3 and caspase 3 in positive regions was quantified by Image J software (NIH, USA).

### Statistical Analysis

Data are represented as mean ± SD. Mann–Whitney *U*-test or one-way ANOVA was used to make comparisons between different groups. Kaplan–Meier method was used to determine mice survival rate. Results were analyzed using SPSS (version 24; Chicago, IL, USA) and GraphPad Prism 6.07 software. Statistical significance threshold was set as *P* < 0.05.

## Results

### Characterization of CT-26 Cell Line-Derived Exosomes

**Figure 2:**
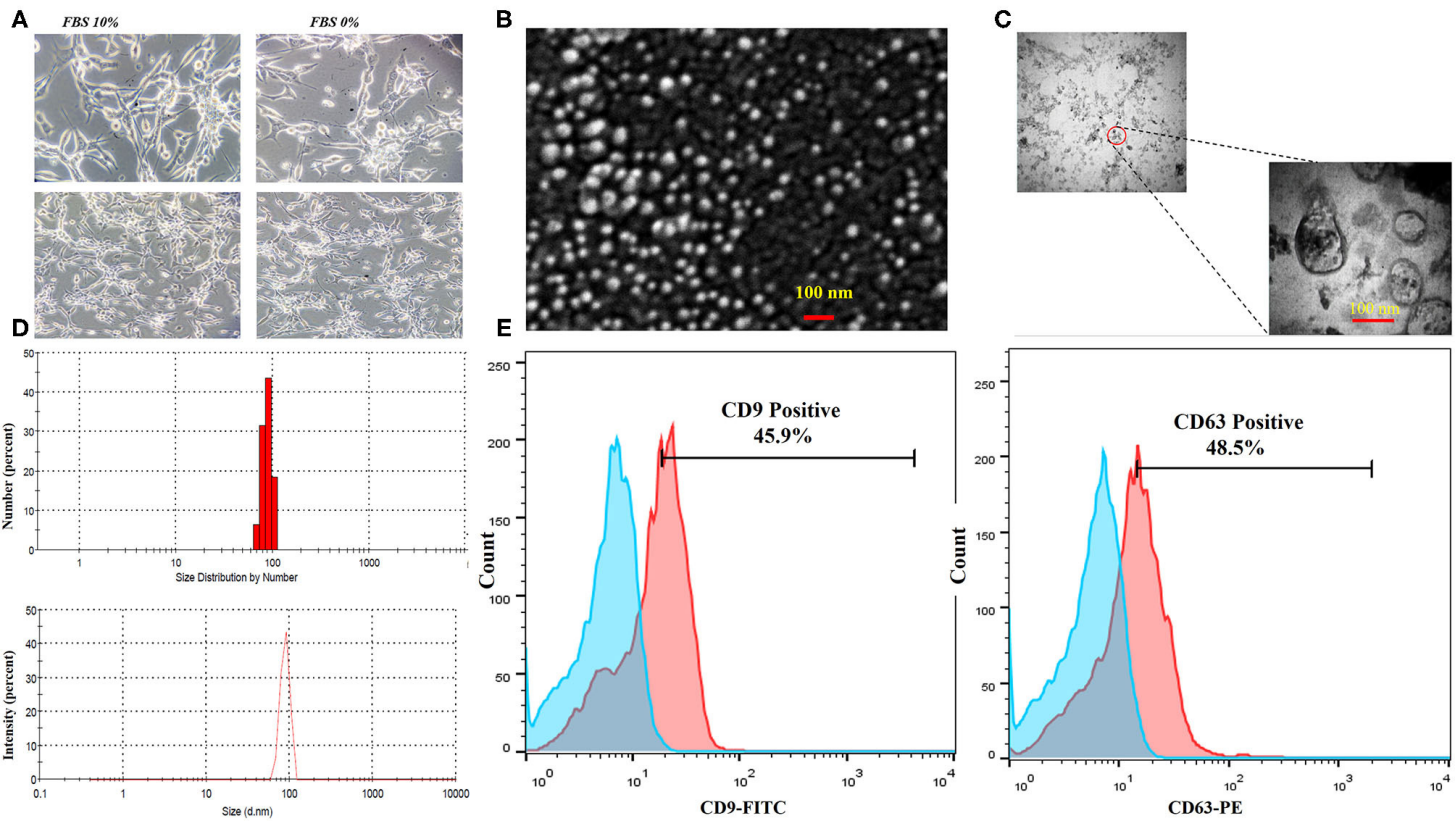
Characterization of CT-26 cell line-derived exosomes. **(A)** Morphology of CT-26 cells in two different conditions, FBS 10% and FBS 0%; in the FBS-free condition under a light microscope. No significant morphology changes were detected. **(B)** Scanning electron microscopic image of CT-26-derived TEXs. **(C)** Transmission electron microscopic image of CT-26-derived TEXs. **(D)** Size distribution of CT-26-derived TEXs by number and intensity using dynamic light scattering. **(E)** Flow cytometry analysis revealed that isolated exosomes were positive for CD9 and CD63 exosomal markers. TEX, tumor exosome.

The CT-26 cells were adapted to FBS-free medium through sequential adaptation (Figure 2A). Based on the BCA method, the average exosome yield was 1,525 μg from 150 ml (5–7 × 10^7^ cells) of CT-26 cell culture supernatant. TEM (Figure 2B) and SEM (Figure 2C) analyses revealed the disc shape of CT-26 cell-derived exosomes and showed that the exosomes had an average size between 30 and 150 nm. Moreover, according to Zetasizer results (Figure 2D), the average size of tumor-derived exosomes was determined to be 95 nm. Analysis of phenotypic markers showed that isolated exosomes were positive for CD9 and CD63 (Figure 2E).

### Efficient Delivery of miR-124-3p Into CT-26-Derived Exosomes

**Figure 3:**
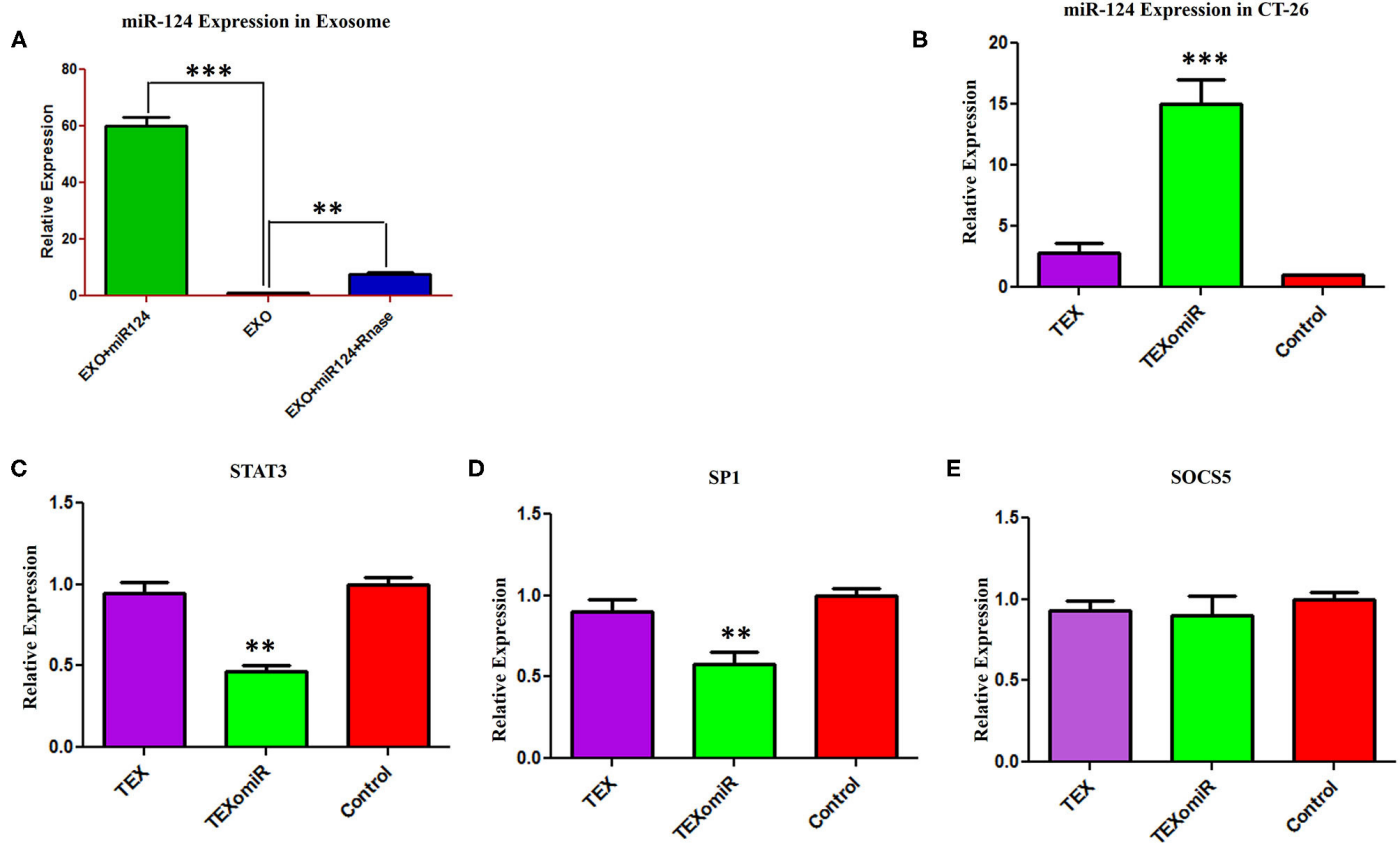
Efficient delivery of miR-124-3p into exosomes and *in vitro* evaluation of the efficacy of miR-124-enriched tumor exosomes (TEXs). **(A)** For delivery of miR-124 mimic into TEX, modified calcium chloride transfection method was applied. To determine the encapsulation efficiency, exosome was reisolated and treated with one unit of RNase A for digestion of unloaded miRNAs. Subsequently, miRNA samples were isolated from purified exosomes, and the expression of miR-124-3p in exosomes was detected using quantitative RT-PCR. U6 snRNA was used as an internal control. **(B)** CT-26 cell lines treated with TEXomiR or TEXs, and the relative expression levels of miR-124 **(C)** STAT-3, **(D)** SP1, and **(E)** SOCS5 were measured using real-time PCR. Successful overexpression of miR-124 and dowregulation of the expression of STAT-3 and SP1 was verified in CT-26 cells that were treated with TEXomiR. The data are presented as mean ± SD. **P < 0.01 and ***P < 0.001.

In order to assess the miR-124-3p delivery potential of CT-26-derived exosomes *in vitro*, exosomes were loaded with miR-124 mimic using the modified calcium chloride transfection method. We purified the miRNAs from exosomes, and then stem-loop RT-PCR reverse transcription was conducted for cDNA synthesis. The results of quantitative real-time PCR determined that the miR-124 expression in miRNA-124 loaded TEXs group compared to unloaded TEX group was significantly upregulated (relative expression: 6.5 ± 0.7 *vs*. 1 ± 0.2; miRNA loading efficiency of 53.2%; *P* < 0.01; Figure 3A). After that, we evaluated the potential efficiency of exosome-mediated miR-124 delivery *in vitro*. Firstly, CT-26 cell lines were seeded at a concentration of 3 × 10^5^ cell/well and then treated with miRNA-loaded exosomes or exosomes alone. Following treatment, the relative expression levels of miR-124 were measured using real time. Successful overexpression of the miR-124 was verified in CT-26 cells that were treated with miR-124-loaded exosomes (Figure 3B). The relative expression of miR-124 in the TEXomiR, TEX, and control groups was 15 ± 3.6, 2.7 ± 1.2, and 1 ± 0.2, respectively (*P* = 0.0023).

It is well-known that miRNAs exert their functions *via* the suppression of their target gene expression. Next, we examined the mRNA expression levels of STAT-3, SOCS-5, and SP-1 as a direct target of miR-124. It was detected that treatment with miR-124-loaded TEX significantly decreased the mRNA expression levels of STAT-3 (Figure 3C) and SP-1 (Figure 3D) in TEXomiR group compared to TEX and control groups (relative expression = 0.46 ± 0.16, *P* < 0.01 and 0.57 ± 0.17, *P* < 0.01, respectively). However, the mRNA expression levels of SOCS-5 did not reach a statistically significant threshold (Figure 3E). Overall, this data represented that miR-124-loaded exosomes were internalized by CT-26 cells successfully.

### Evaluation of the Cytotoxic Effect of miRNA-Enriched Exosomes

**Figure 4:**
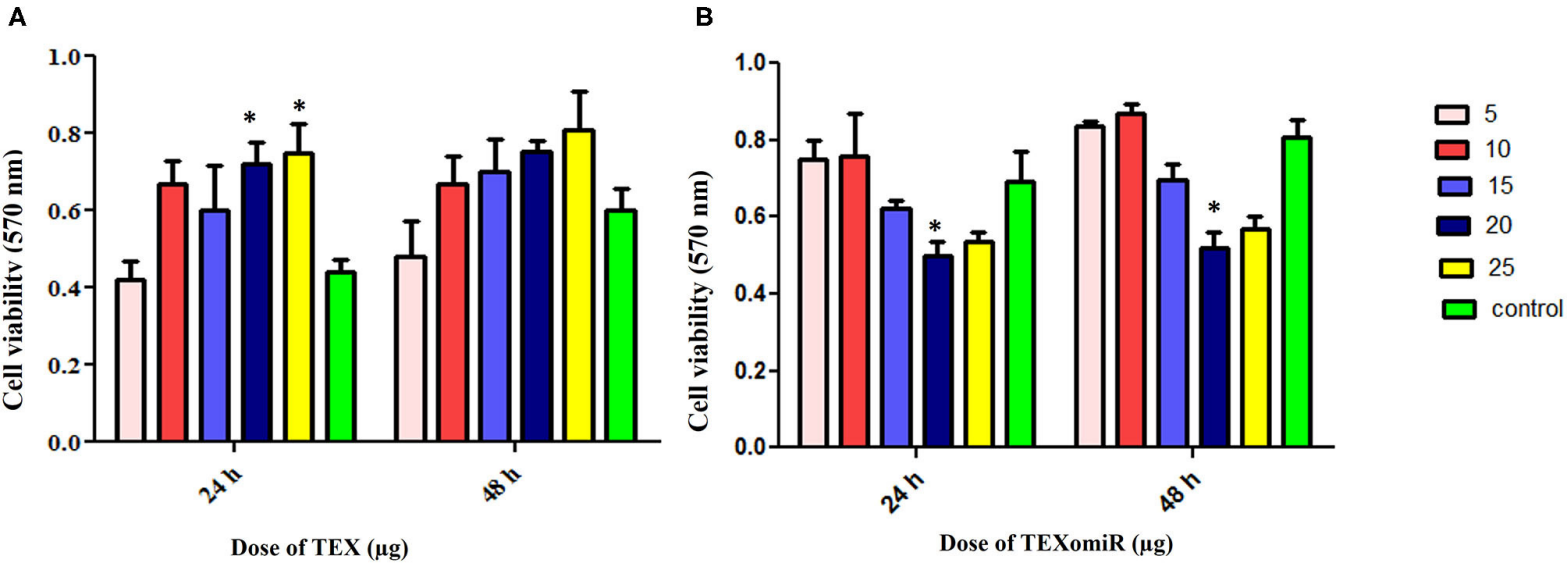
Evaluation of the cytotoxicity effect of TEXomiR delivery on CT-26 cells. **(A)** Proliferation and cell viability were significantly increased by tumor exosome in a dose-dependent manner. **(B)** TEXomiR showed significant cytotoxicity at the dose of 20 and 25 μg. The data are representative of three independent experiments and presented as mean ± SD. *P < 0.05 *vs*. untreated or control group.

The cytotoxicity of the unloaded TEX and miR-124-3p-enriched TEX (TEXomiR) on CT-26 cells was investigated by MTT assay after 24 and 48 h in five concentrations (5, 10, 15, 20, and 25 μg). Cell proliferation and viability of CT-26 cells treated with TEX were significantly increased compared to the control (untreated cells) (Figure 4A). At the time point of 24 and 48 h, the highest proliferation was observed at doses of 20 and 25 μg compared to the control group, respectively (*P* < 0.05) (Figure 4A). On the other hand, the cell viability of CT-26 cells treated with TEXomiR was moderately decreased compared to the control group probably due to the delivery of miR-124-3p to CT-26 cells. At the time point of 24 and 48 h, the highest cytotoxicity was observed at the dose of 20 μg compared to the control group (*P* < 0.05) (Figure 4B).

**Figure 5:**
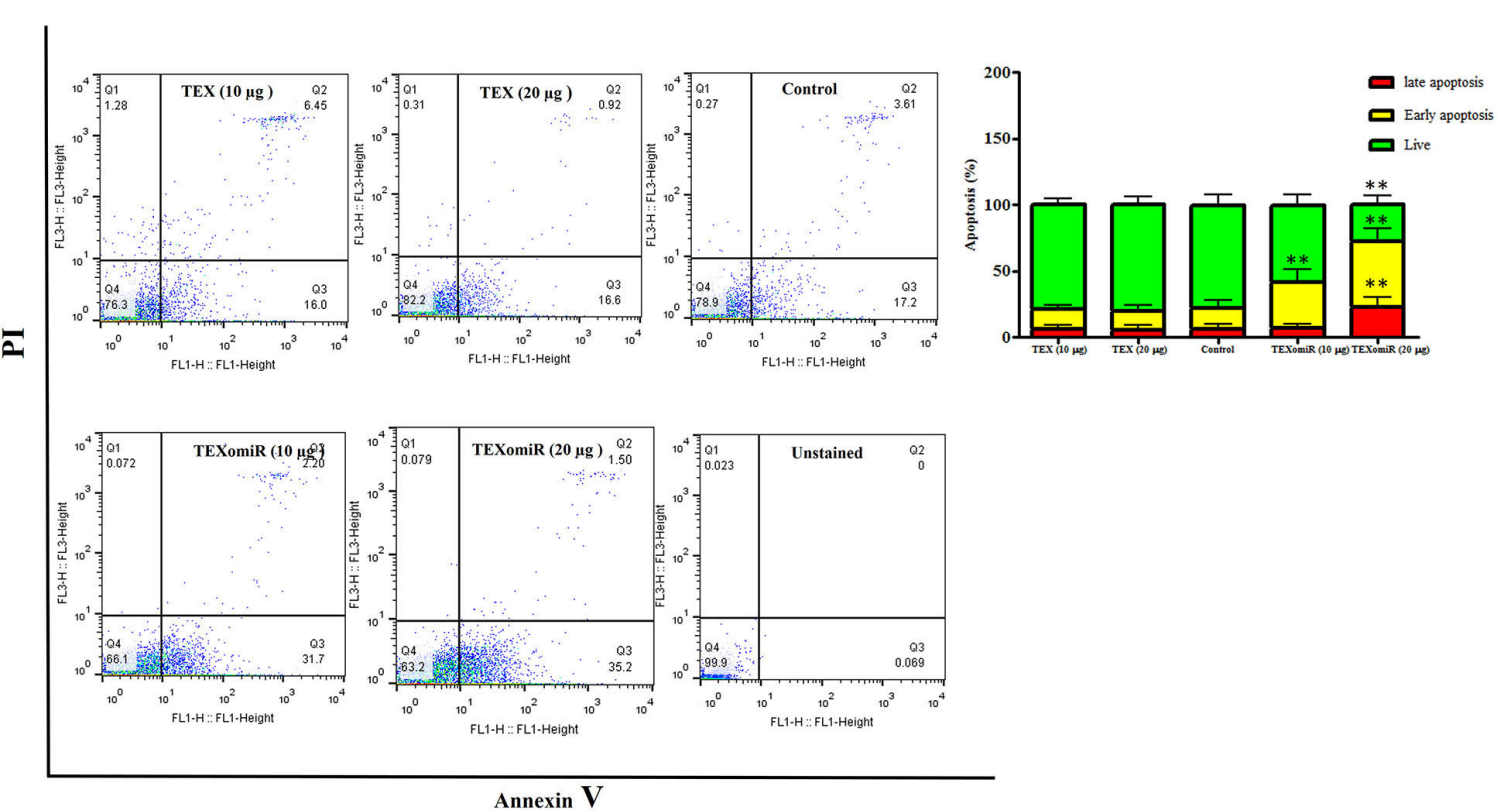
The effect of TEXomiR on cell apoptosis. TEX had no significant effects on the apoptosis of CT-26 cells. Following treatment with TEXomiR, the apoptosis percentage of CT-26 cells significantly increased. The data are representative of three independent experiments presented as mean ± SD. **P < 0.01. One-way ANOVA was used to make comparisons between groups.

The effect of TEXomiR and TEX (10 and 20 μg) treatment on the apoptosis of CT-26 cells after 48 h is presented in Figure 5. For assessing the apoptosis levels of CT-26 cells, the FITC Annexin V/PI method was conducted. As summarized in Figure 5, TEX treatment had no significant effects on apoptosis percentage of CT-26 cells compared to the untreated cells [apoptosis%: TEX (10 μg) = 21.4, TEX (20 μg) = 19.5, control = 21.5]. On the other hand, the apoptosis percentage of CT-26 cells was significantly augmented following treatment with TEXomiR compared to TEX (*P* < 0.01) and untreated cells (*P* < 0.01) in dose-dependent manner [apoptosis%: TEXomiR (10 μg) = 41.3, TEXomiR (20 μg) = 72.1, control = 21.5] (Figure 5).

### *In vivo* Assay: Inhibition of Tumor Growth by Immunization With TEXomiR

**Figure 6:**
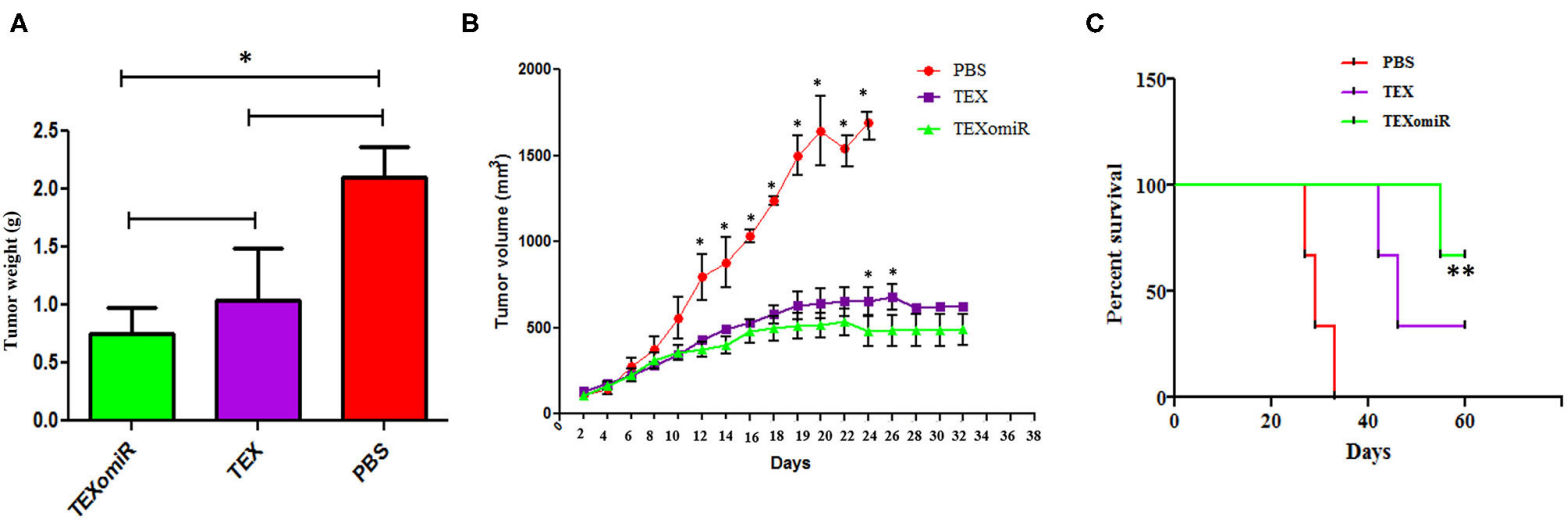
The anti-tumor efficiency of TEXs and TEXomiR in female BALB/c mice bearing CT-26 colon tumor. **(A)** The tumor weights in mice treated with TEXomiR were significantly different from those of phosphate-buffered saline (PBS)-treated mice. **(B)** Inoculation with TEX or TEXomiR significantly inhibits tumor growth. **(C)** The mice treated with TEXomiR had a significantly longer survival rate than PBS-treated mice or those treated with TEXs. The data are presented as mean ± SD. **P < 0.01, *P < 0.05. One-way ANOVA was used to make comparisons between groups. TEXomiR, miR-124-3p-enriched TEXs; TEXs, tumor exosomes.

The actual tumor weights in mice treated with TEXomiR and TEX were slightly different (0.75 ± 0.2 *vs*. 1.03 ± 0.5 g, respectively) (*P* > 0.05). However, in the mice treated with PBS, actual tumor weight was significantly increased (2.1 ± 0.3 g) compared with the TEXomiR group (*P* = 0.032) (Figure 6A). As presented in Figure 6B, the tumor grows and develops quickly in control mice injected with PBS. Treatment with TEX or TEXomiR significantly inhibits tumor growth in comparison with the PBS group (Figure 6B). The mean tumor volume in the TEXomiR group was significantly lower on days 14, 16, 18, 20, 22, 24, 26, and 28 compared to the PBS group (*P* < 0.001). Moreover, a significant difference in tumor volume was observed between the TEXomiR and TEX groups in days 24 and 26 (*P* = 0.028). Survival analyses revealed that the CT-26 tumor-bearing mice treated with TEXomiR had a considerably longer mean survival (ms) (ms = 60.0 days) than TEX-treated mice (ms = 46 days) or mice treated with PBS (ms =29 days) (*P* = 0.0051) (Figure 6C).

### Evaluation of Tumor-Infiltrating and Lymph Node Lymphocytes

**Figure 7:**
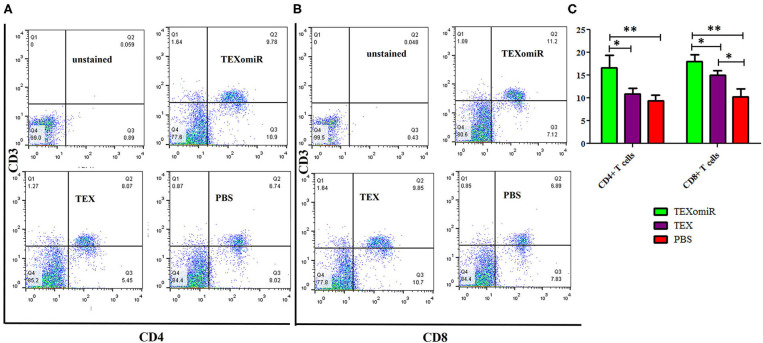
The effect of TEXomiR on lymph node lymphocytes. **(A,B)** Flow cytometry analysis of CD4 T+ and CD8 T+ lymphocytes in lymph node from CT-26 tumor-bearing mice treated with TEXomiR, TEXs, and phosphate-buffered saline (PBS). **(C)** A significant increase in the number of CD8+ and CD4+ T lymphocytes was detected in the TEXomiR group compared with the PBS group. The data are presented as mean ± SD. **P < 0.01, *P < 0.05. One-way ANOVA was used to make comparisons between groups. TEXomiR, miR-124-3p-enriched TEXs; TEX, tumor exosomes.

**Figure 8:**
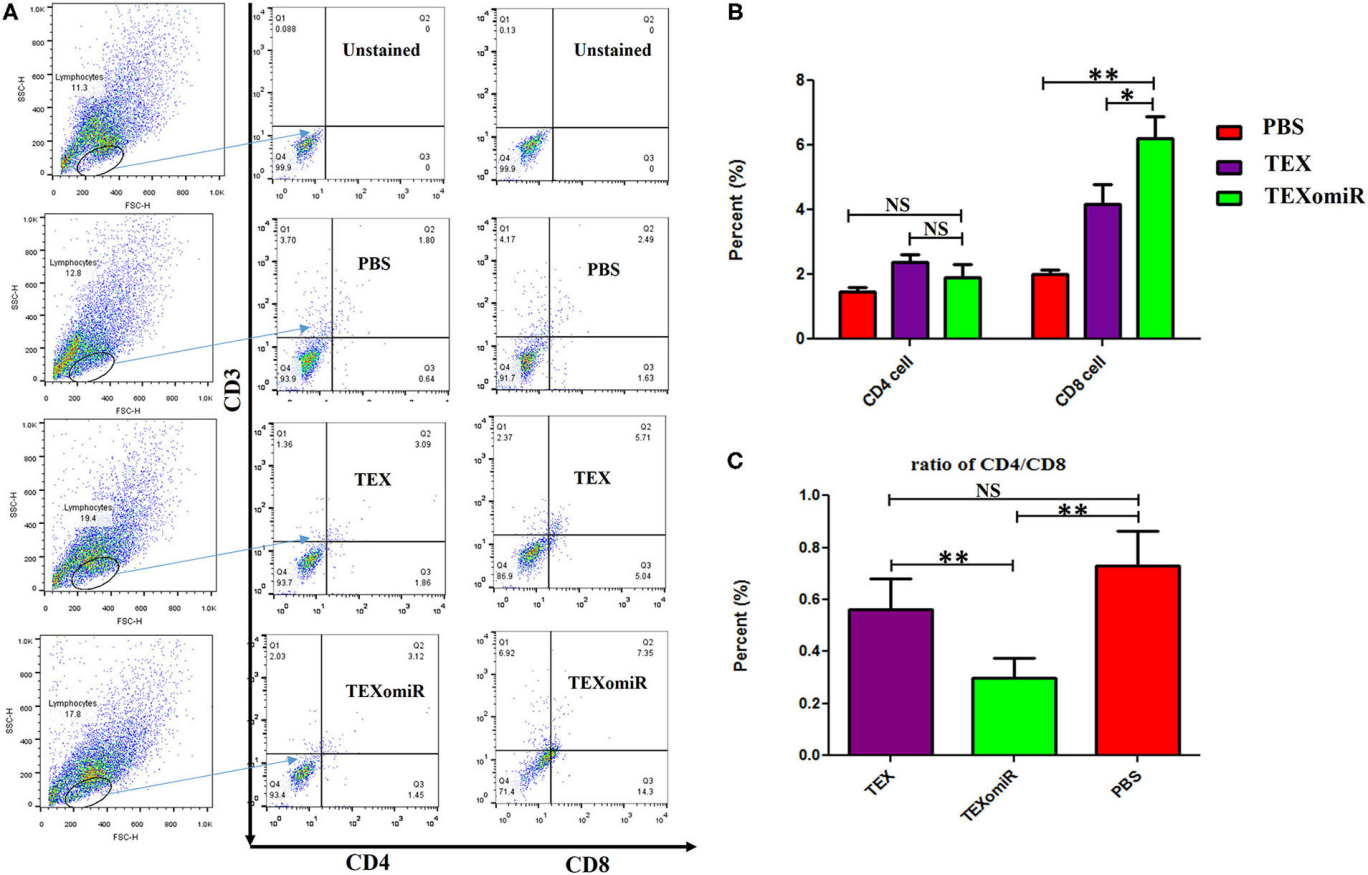
The effect of TEXomiR on tumor lymphocytes. **(A)** Flow cytometry analysis of CD4 T+ and CD8 T+ lymphocytes in tumor from CT-26 tumor-bearing mice treated with TEXomiR, TEXs, and phosphate-buffered saline (PBS). **(B)** A significant increase in the number of CD8+ T lymphocytes was detected in the TEXomiR group compared with the TEXs and PBS groups. **(C)** The ratio of CD4/CD8 showed a significant decrease in the TEXomiR group compared with the TEXs and PBS groups. The data are presented as mean ± SD. **P < 0.01, *P < 0.05. One-way ANOVA was used to make comparisons between groups. TEXomiR, miR-124-3p-enriched TEXs; TEX, tumor exosome.

The effect of TEXomiR on tumor-infiltrated and lymph node lymphocytes was assessed by flow cytometry. The results illustrated a significant (*p* < 0.05) increase in the CD4^+^ T cells of lymph nodes (Figures 7A–C) in the TEXomiR group compared with the TEX (*P* = 0.021) and PBS-treated (*P* = 0.0069) groups. The frequency of CD4^+^ T cells in TEXomiR, TEX, and PBS groups was 16.67 ± 4.6, 10.94 ± 2.6, and 9.37% ± 2.5, respectively. The frequency of CD8^+^ T cells in TEXomiR-treated mice compared to the TEX (*P* = 0.045) and PBS (*P* = 0.0012) groups was significantly increased (18 ± 2.8 *vs*. 15% ± 1.8 and 10.3% ± 3.4, respectively). Furthermore, a significant increase in the CD8+ subpopulation of lymph node lymphocytes of mice treated with TEX compared with the PBS group has been demonstrated (15 ± 1.8 *vs*. 10.3% ± 3.4) (*p* < 0.05). On the other hand, tumor-infiltrated lymphocyte (TIL) analysis revealed a significant increase in CD8^+^ T cells in the TEXomiR group compared to TEX (*P* = 0.039) and PBS-treated (*P* = 0.0063) mice (6.2 ± 1.13 *vs*. 4.17 ±1.23 and 2% ±1.1) (Figures 8A,B). There was no significant difference in the frequency of CD4+ subpopulation of TILs between the TEXomiR group and the TEX or PBS groups (Figures 8A,B). Finally, a significant decrease in the CD4+/CD8+ ratio (Figure 8C) in mice treated with TEXomiR compared with both TEX (*P* = 0.0087) and PBS-treated (0.0038) mice was observed (0.3 ± 0.13 *vs*. 0.56 ± 0.21 and 0.73% ± 0.071).

### Evaluation of the Tumor-Infiltrating CD4+CD25+Foxp3+ Regulatory T Cells

**Figure 9:**
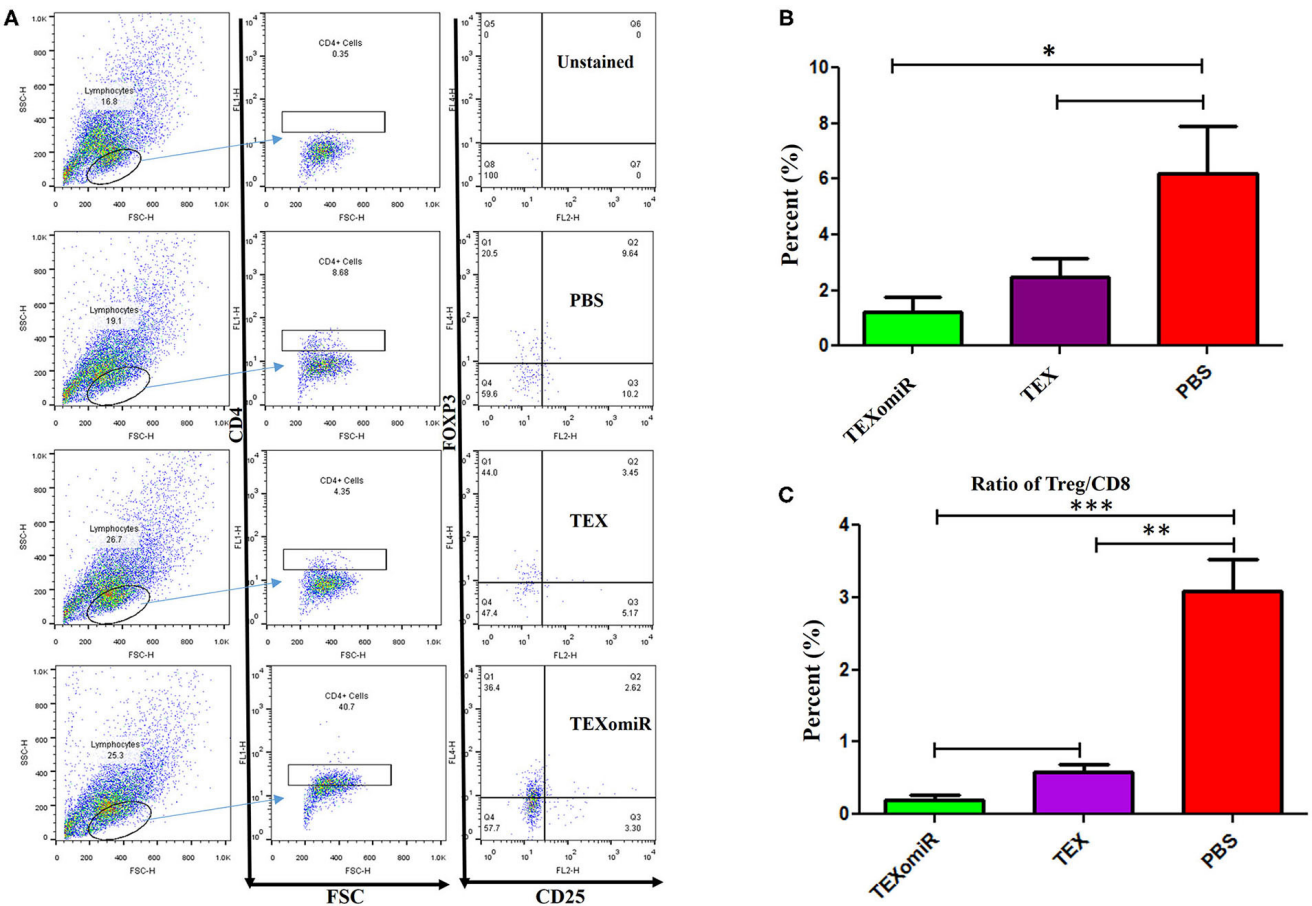
The effect of TEXomiR on tumor-infiltrating CD4+CD25+Foxp3+ regulatory T cells. **(A)** Flow cytometry analysis of tumor-infiltrating CD4+CD25+Foxp3+ regulatory T cells in tumor from CT-26 tumor-bearing mice treated with TEXomiR, TEXs, and phosphate-buffered saline (PBS). **(B)** A significant decrease in the number of tumor-infiltrating CD4+CD25+Foxp3+ regulatory T cells was detected in the TEXomiR group compared with the PBS group. **(C)** The ratio of Treg/CD8+ showed a significant decrease in mice treated with TEXomiR compared with the TEX and PBS-treated mice. The data are presented as mean ± SD. *P < 0.05, **P < 0.01, ***P < 0.001. One-way ANOVA was used to make comparisons between groups. TEXomiR, miR-124-3p-enriched TEXs; TEX, tumor exosome.

Analysis of the effect of TEXomiR on the frequency of tumor-infiltrated CD4+CD25+Foxp3+ regulatory T cells was conducted by flow cytometry in a CD4+ T cell population. The evaluation of tumor-infiltrated CD4+CD25+Foxp3+ regulatory T cells designated that treatment of mice with TEXomiR significantly (*P* = 0.032) decreases the tumor-infiltrated CD4+CD25+Foxp3+ T cells in the TEXomiR group compared with the PBS group (Figures 9A,B). The frequency of regulatory T cells (Treg) in the TEXomiR, TEX, and PBS groups was 1.2 ± 1.1, 2.48 ± 1.37, and 6.17% ± 3.47, respectively. Moreover, the results determined a slight decrease in tumor-infiltrating CD4+CD25+Foxp3+ T cells in the TEXomiR group compared with TEX; however, it did not reach a statistically significant threshold (*P* = 0.071) (Figure 9B). Subsequently, the ratio of Treg to CD8+ Tcell (Treg/CD8+ ratio) represented a significant decrease in mice treated with TEXomiR (*P* = 0.00026) and TEX (*P* = 0.0089) compared with PBS-treated mice (Figure 9C). The ratio of Treg to CD8+ Tcell in the TEXomiR, TEX, and PBS groups was 0.19 ± 0.12, 0.58 ± 0.2, and 3.09% ± 0.75, respectively.

### Cytotoxic Activity of Splenocytes

**Figure 10:**
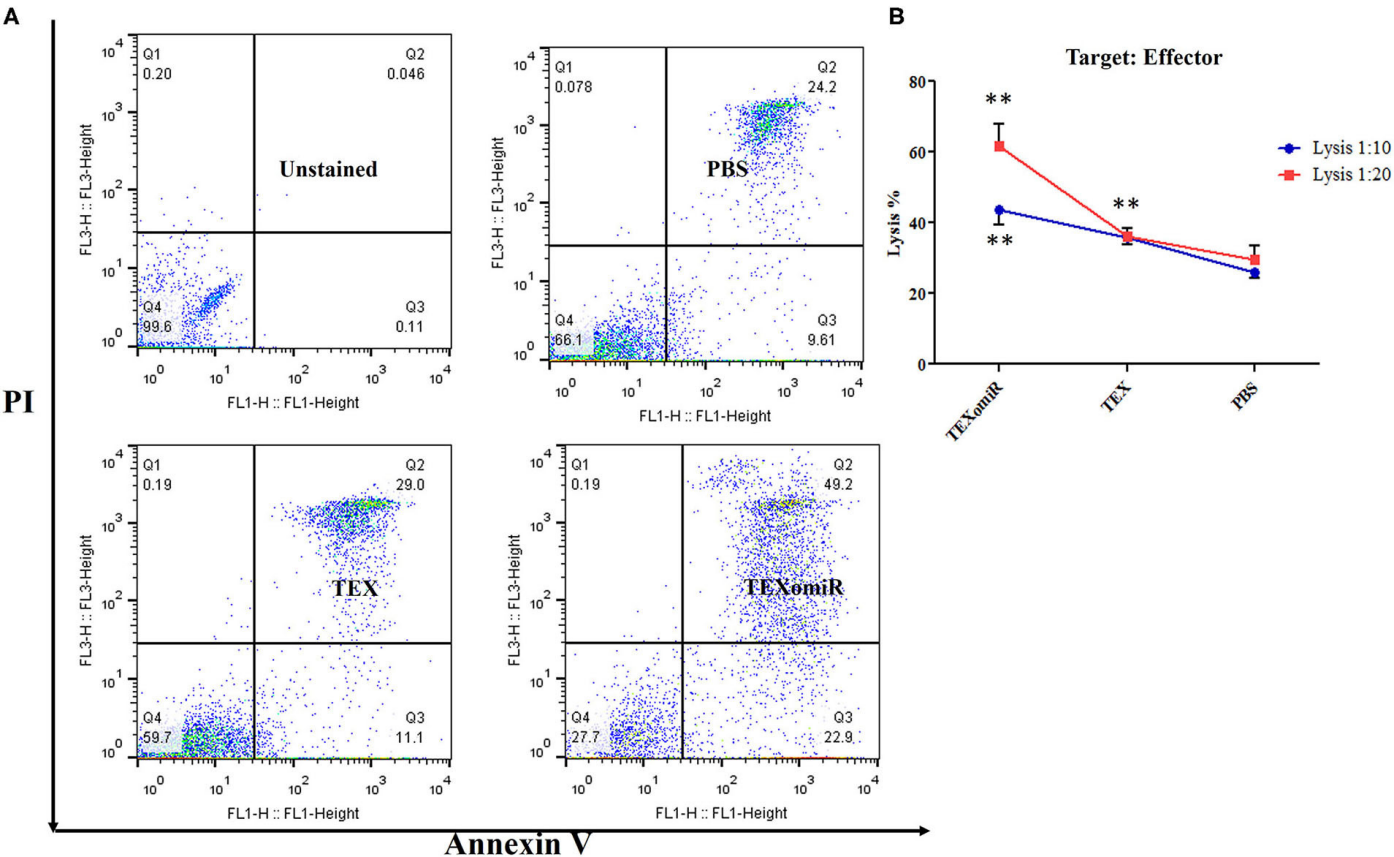
Evaluation of the cytotoxic activity of splenocytes. **(A)** The TEXomiR group displayed a significant raise in cytotoxic activity against the target CT-26 cells compared with the TEX and phosphate-buffered saline (PBS) groups. **(B)** Lysis percentage in TEXomiR, TEX, and PBS groups at the ratio of 1:10 and 1:20. The data are presented as mean ± SD. **P < 0.01. One-way ANOVA was used to make comparisons between groups. TEXomiR, miR-124-3p-enriched TEXs; TEX, tumor exosome.

To ascertain whether repeated injection of TEXomiR could stimulate a robust CTL response, splenocytes were collected 2 weeks after the last injection. The cytotoxic activity of the splenocytes was conducted by the FITC Annexin V method. The splenocytes from the mice in the TEXomiR group displayed a significant raise in cytotoxic activity against the target CT-26 cells compared with both the TEX and PBS groups (Figure 10A). The apoptosis percentages of TEXomiR, TEX, and PBS groups were as follows: at a ratio of 1:10, 43.6 ± 8.1, 35.9 ± 4.01, and 26% ± 3.2, respectively (*P* = 0.0053), and at a ratio of 1:20, 61.8 ± 10.6 *vs*. 36.7 ± 4.6 and 30.1% ± 7.3, respectively (Figure 10B). The cytotoxic activity of splenocytes in mice treated with TEXomiR showed a significant difference compared with PBS-treated mice at the ratio of 1:10 (*P* = 0.0053). On the other hand, at the ratio of 1:20, there was a significant difference in the cytotoxic activity of the TEXomiR group compared with both the TEX and PBS groups (*P* = 0.0041, *P* = 0.0023).

### Relative Expression of SP1, SOCS5, and STAT3 in the Splenocytes of TEXomiR-Treated Mice

**Figure 11:**
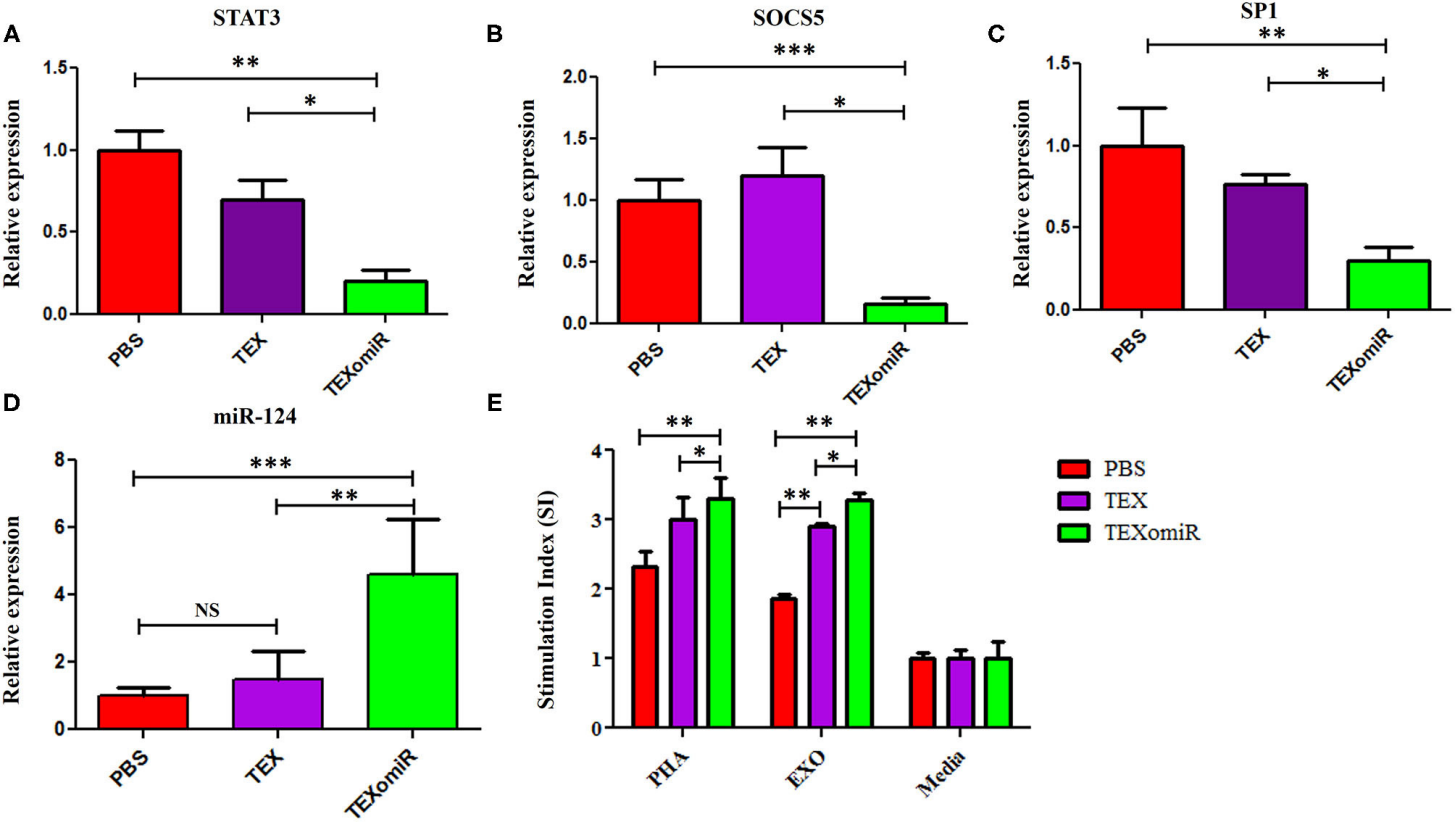
The effect of TEXomiR on miR-124 target genes and lymphocyte proliferation in splenocytes from CT-26 tumor-bearing mice. **(A)** qRT-PCR analysis represented a significant reduction of STAT3 expression in the TEXomiR group compared to the TEX and phosphate-buffered saline (PBS) groups. **(B)** Relative expression of SOCS5 in the TEXomiR, TEX, and PBS groups. **(C)** Relative expression of SP1 in the TEXomiR, TEX, and phosphate-buffered saline (PBS) groups. **(D)** Relative expression of miR-124 in splenocytes of TEXomiR, TEX, and PBS-treated mice. **(E)** MTT test for evaluation of the stimulation index of lymphocytes in the spleen. The data are presented as mean ± SD. *P < 0.05, **P < 0.01, ***P < 0.001. One-way ANOVA was used to make comparisons between groups. TEXomiR, miR-124-3p-enriched TEXs; TEX, tumor exosome.

The effect of the administration of TEXomiR, TEX, and PBS on the relative expression of the miR-124 target genes including STAT3 (Figure 11A), SOCS5 (Figure 11B), and SP1 (Figure 11C) was measured by real-time PCR. The results indicated a significant reduction of STAT3, SP1, and SOCS5 mRNA expression in the splenocytes of CT-26 tumor-bearing mice treated with TEXomiR compared to both the TEX and PBS groups (*P* < 0.01). The relative expression of STAT3, SOCS5, and SP1 in the TEXomiR group compared to the TEX and PBS groups were as follows: (0.2 ± 0.11 *vs*. 0.7 ± 0.23 and 1 ± 0.2, *P* = 0.0029), (0.16 ± 0.09 *vs*. 1.2 ± 0.4 and 1 ± 0.3, *P* = 0.00089), and (0.3 ± 0.15 *vs*. 0.77 ± 0.1 and 1 ± 0.4, *P* = 0.0073), respectively. There was no significant difference between the TEX and PBS groups. Moreover, an increased expression of miR-124 was represented in splenocytes of TEXomiR-treated mice compared to both the TEX and PBS groups (Figure 11D). The relative expression of miR-124 in the TEXomiR group compared to the TEX and PBS groups was as follows: (4.6 ± 1.6 *vs*. 1.5 ± 0.81 and 1 ± 0.24, *P* = 0.0063 and *P* = 0.00011).

### Lymphocyte Proliferation Test

MTT test was used to evaluate the proliferative capacity of lymphocytes in the spleen. The results of the MTT test indicate that treatment with PHA induces a significant increase in the stimulation index of the lymphocytes in the TEXomiR group compared with the TEX and PBS groups (*P* < 0.05). Moreover, treatment with tumor exosome (EXO) showed a significant increase in the stimulation index of the lymphocytes in the TEXomiR and TEX groups compared with the PBS group (*P* = 0.0012, *P* = 0.031). In addition, the stimulation index difference between the TEXomiR and TEX groups was statistically significant (*P* = 0.029) (Figure 11E). There was no significant difference between the PHA-treated groups and the EXO-treated groups. The mean stimulation index of the PHA- and EXO-treated groups was 8.64 ± 1.5 *vs*. 8.05 ± 0.4, respectively (*P* = 0.21).

### Elevated Th1 Cytokine Production in TEXomiR-Treated Mice Splenocytes

**Figure 12:**
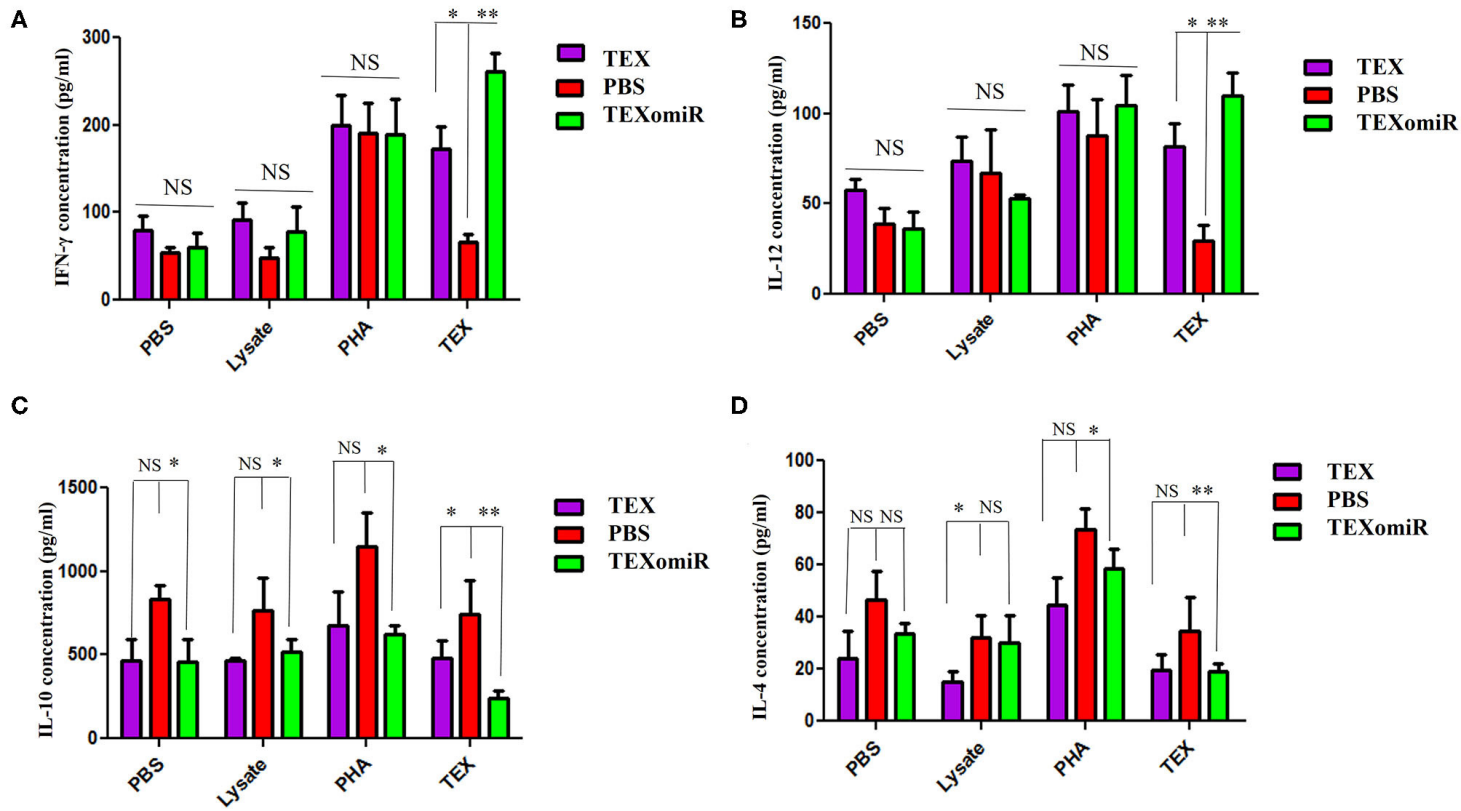
IFN-γ **(A)**, IL-12 **(B)**, IL-10 **(C)**, and IL-4 **(D)** production by splenocytes from tumor-bearing mice in different groups. The splenocytes were cultured *in vitro* with TEX, phytohemagglutinin, tumor lysate, and media. The results are shown as mean ± SD (*n* = 8 mice per group). *P < 0.05, **P < 0.01. One-way ANOVA was used to make comparisons between groups.

The splenocytes from the mice were assessed for the production of IL-12, IFN-γ, IL-4, and IL-10. In the TEXomiR groups, analysis of cytokine profile from spleen cells treated with TEX indicated a significant increase in IFN-γ and IL-12, a signature cytokine of the Thl immune response, compared to the TEX and PBS groups (27). The mean concentration of IFN-γ and IL-12 in the TEXomiR, TEX, and PBS groups was as follows: (260.5 ± 11.3 *vs*. 172.75 ± 9.6 and 66.5 ± 6.3, *P* < 0.05) and (109.75 ± 8.3 *vs*. 81.5 ± 7.4 and 29.25 ± 5.3, *P* < 0.05), respectively. There was no significant difference in the mean concentration of IFN-γ and IL-12 from spleen cells treated with tumor lysate and PHA in the TEXomiR, TEX, and PBS groups (Figures 12A,B). On the other hand, a significant decrease in tumor-progressive cytokines, including IL-10 and IL-4, from spleen cells treated with TEX was identified in the TEXomiR group compared to both the TEX and PBS groups (Figures 12C,D). The mean concentration of IL-10 and IL-4 in the TEXomiR, TEX, and PBS groups was as follows: (243 ± 16.3 *vs*. 479 ± 29.3 and 743 ± 73.2, *P* < 0.05) and (19.25 ± 4.3 *vs*. 19.5 ± 5.2 and 34.5 ± 12.3, *P* < 0.05), respectively. These results indicated that TEXomiR changed the immune milieu from immunoinhibitory to immunostimulatory, and it is essential for the prognosis of CT-26 tumor.

### Histopathological Findings

**Figure 13:**
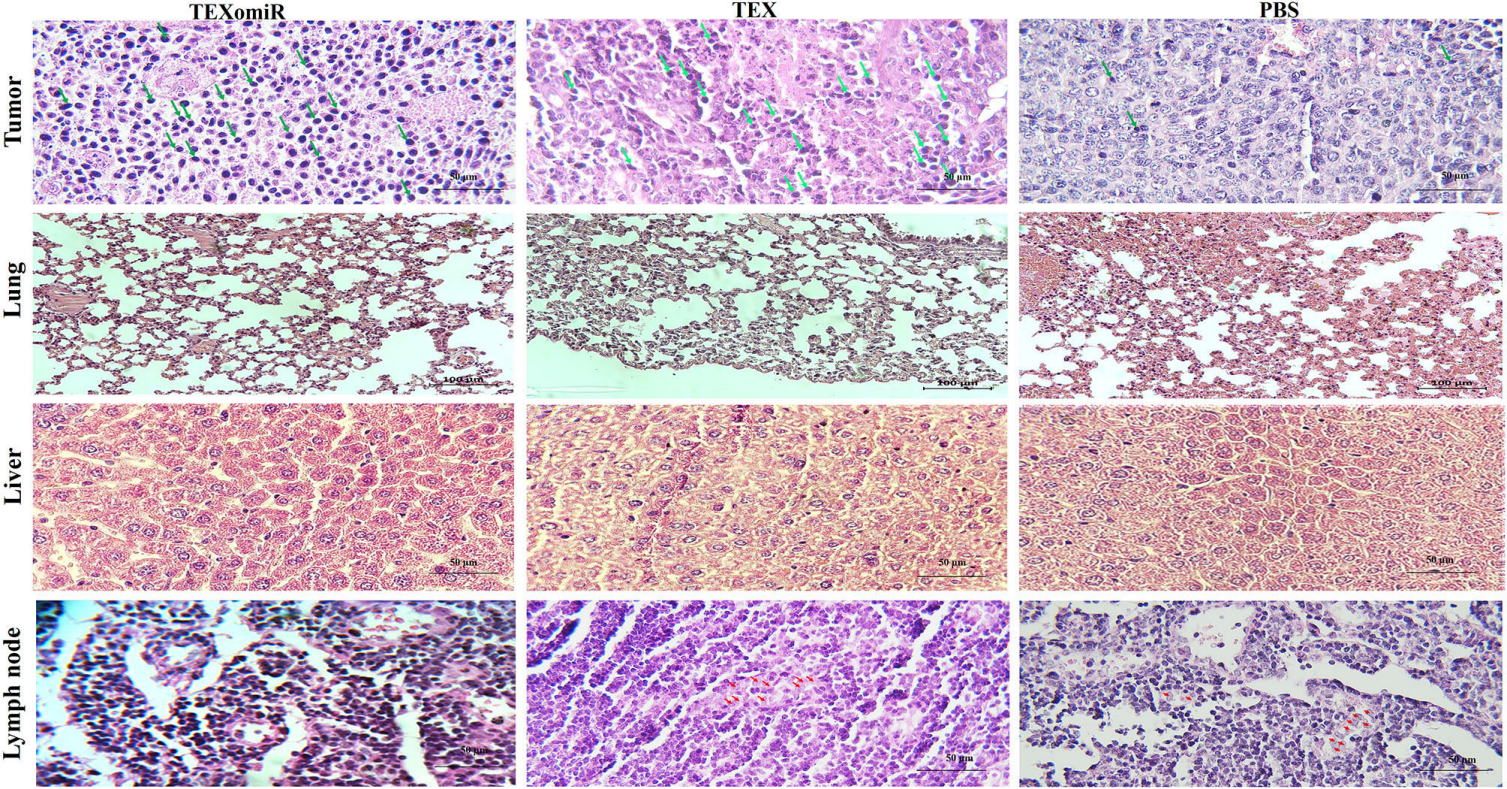
Histopathological results of tumor, lymph node, lung, and liver tissues from CT-26 tumor-bearing mice in different groups. Lymphocyte infiltration in tumor microenvironment (magnification, ×400) and tumor metastasis to lung (magnification, × 200), liver (magnification, × 400), and lymph nodes (magnification, × 400) in the TEXomiR, TEX, and PBS groups. The arrow indicates lymphocyte infiltration in tumor tissue and metastasis to lymph node. The green arrows indicate lymphocyte infiltration, while the red arrows indicate metastasis.

H&E staining of tumor sections determined that tumors related to the PBS group showed a higher grade. Moreover, the PBS group showed significant cellular atypism with severe pleomorphism and mitotic activity. The treatment with TEXomiR and TEX revealed a wide area of apoptosis, necrosis, and regression of tumor. The histopathological results of lymph nodes showed that, except for the TEXomiR group, initial tumor metastasis to lymph nodes was identified in both the PBS and TEX groups (Figure 13). As presented in Figure 13, CT-26 tumor-bearing mice treated with PBS, compared to the TEXomiR and TEX groups, presented significant histopathological abnormalities in the lung, including infiltration of inflammatory cells, bronchial fibrosis, and pulmonary congestion. However, no considerable tumor metastasis to the lung was observed in any of the three groups. While lymphocyte infiltration into the tumor microenvironment was mild and moderate in the PBS and TEX groups, the TEXomiR group showed a considerably high lymphocyte infiltration (Figure 13). Finally, the TEXomiR and TEX groups showed high and moderate tumor apoptosis, respectively; the PBS group did not show considerable tumor apoptosis (Supplementary Figure 1).

### The Results of Immunostaining of CD3 and Caspase 3 in Tumor

**Figure 14:**
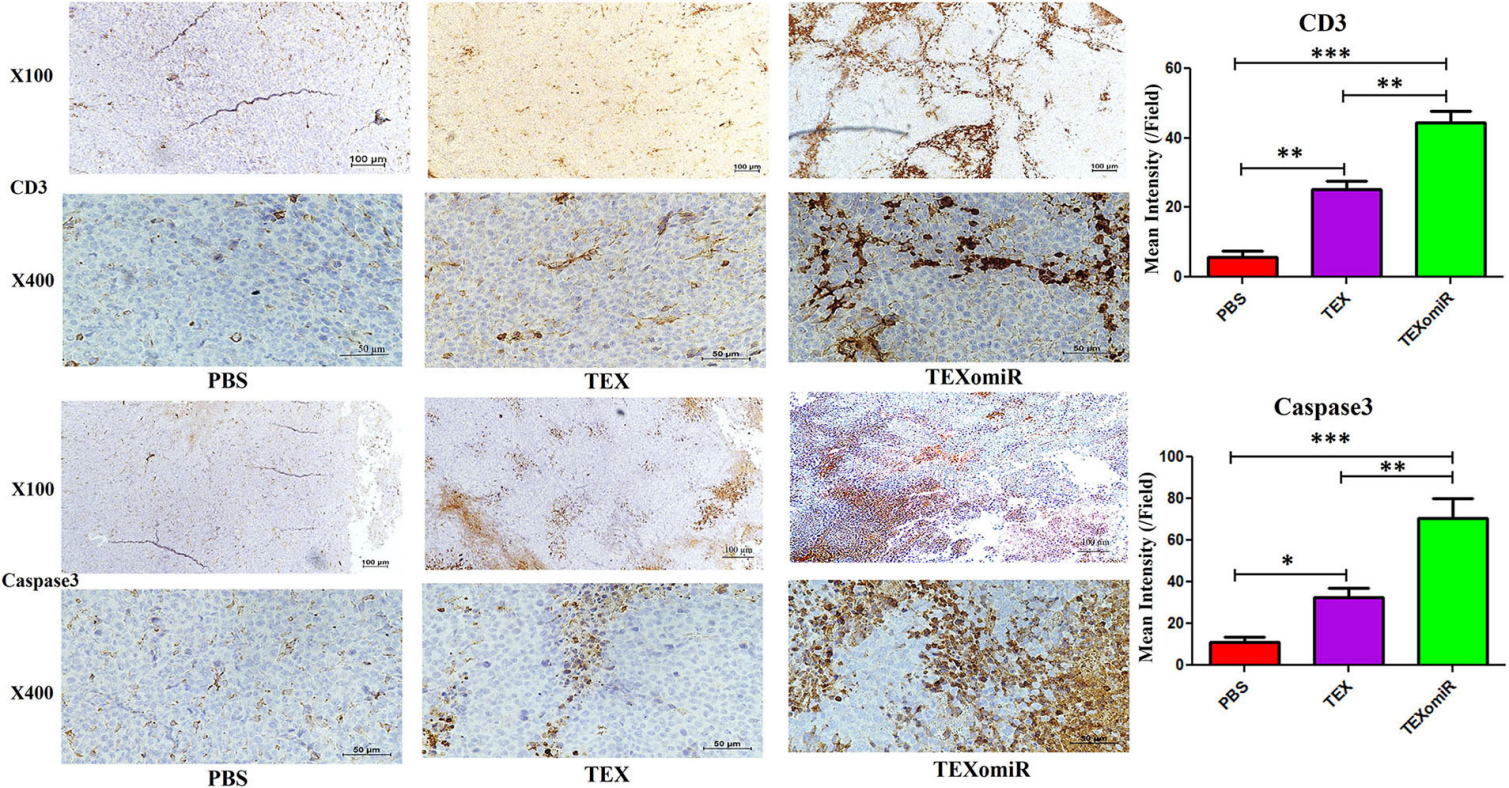
Immunohistochemistry results of the tumor tissues of CT-26 tumor-bearing mice. Treatment with TEXomiR significantly increased tumor-infiltrated lymphocytes compared to the TEX and phosphate-buffered saline (PBS) groups. Microscopic observation also clarifies the high expression of caspase 3 in the tumor sections of TEXomiR-treated mice. The TEXomiR-treated group significantly showed a higher mean intensity of CD3 and caspase 3 compared to the TEX and PBS groups. While the immunoreactivity of CD3 and caspase 3 in the TEXomiR group was diffuse, the TEX and PBS groups represented a scattered pattern. The images were quantified by Image J software (NIH, USA). The data are presented as mean ± SD. *P < 0.05, **P < 0.01, ***P < 0.001. One-way ANOVA was used to make comparisons between groups. TEXomiR, miR-124-3p-enriched TEXs; TEX, tumor exosome.

To validate lymphocyte infiltration and tumor apoptosis in the histopathological findings, we applied IHC for the expression of CD3 and caspase 3 on a range of tumor sections in the TEXomiR, TEX, and PBS groups. IHC analysis of the tumor tissues demonstrated that the expression of CD3, a pan-marker of T lymphocytes, significantly increased in TEXomiR-treated mice compared to the TEX and PBS groups. Moreover, there was a significant difference between the TEX and PBS groups. In the TEXomiR group, immunoreactivity and intensity of CD3 was diffuse and strong, respectively. The mean intensity of CD3 in tumor sections of the TEXomiR, TEX, and PBS groups was as follows: (44.3 ± 6.11 *vs*. 25.4 ± 3.8 and 5.7 ± 3.5, *P* < 0.01), respectively (Figure 14). In addition, IHC analysis of the tumor slices determined that the expression of caspase 3, as a marker of apoptosis, significantly increased in the TEXomiR group compared to the TEX and PBS groups. Moreover, the activity of caspase 3 in TEX-treated mice was significantly higher than that of the PBS-treated group. The mean intensity of caspase 3 in tumor samples of the TEXomiR, TEX, and PBS groups was as follows: (70.4 ± 16.6 *vs*. 32.7 ± 7.6 and 11.5 ± 3.8, *P* < 0.01), respectively (Figure 14).

## Discussion

miRNA replacement therapy compensates the decreased expression of miRNAs with the sequence of oligonucleotide mimics similar to the sequence of the mature endogenous miRNA, previously described as miRNA mimics (28). To achieve a similar biological function as the naturally synthesized miRNA, mimics should have the capability to enter the RNA-induced silencing complex and affect mRNAs as direct target of miRNA (27). Accordingly, a single-strand RNA molecule possesses the same sequence as the mature miRNA and would function as a miRNA mimic. In contrast to single-stranded miRNA mimics, double-stranded miRNA mimics, which are composed of a passenger strand and a guide strand, have 100–1,000-fold higher potency (29). The passenger strand sequence is complementary to the mature miRNA, and the guide strand comprises a sequence like the mature miRNA (30).

In the present study, we observed that tumor-derived exosomes with immunogenic properties can be used to efficiently deliver functionally active miRNA for cancer immunotherapy. Our hypothesis was that administration of TEXomiR as a cell-free cancer vehicle improves anti-tumor immunity in CT-26 mouse tumor models. In order to load the exosomes with miR-124-3p, a modified calcium chloride method was used, and the encapsulation efficiency in the loaded exosomes was approved by real-time PCR.

The evaluation of tumor volume during the study showed that the highest growth rate was identified in the PBS group. The mean tumor volume in the TEXomiR group was significantly lower on days 14–28 than the tumor volume in the PBS group (*P* < 0.001). The mean tumor volume between the TEXomiR and TEX groups on days 24 and 26 showed a statistically significant difference, *P* < 0.05 (Figure 5). While significant tumor growth inhibition was demonstrated with TEXomiR and TEX *in vivo*, no complete tumor eradication was identified in our study, highlighting the tenacity of CT-26 tumor and colorectal cancer at overcoming current treatments. The delivery routes and dose-dependent regimens are also likely to influence the immunotherapeutic effect of TEXomiR; accordingly, there is more room for optimization. In this study, a subcutaneous injection of TEXs showed promising results in reducing tumor growth and stimulating the anti-tumor immune response. Actually, the subcutaneous delivery of TEXs seems to lead to uptake by CD11c+ DCs that eventually migrate to the draining lymph node (31). These results suggest that the context in which the immune system encounters TEX is essential in determining immunosuppression *vs*. immune stimulation.

Moreover, a significant (*p* < 0.05) increase in the CD4+ and CD8+ T cells of tumor-infiltrated and lymph node lymphocytes in the TEXomiR group compared with the PBS-treated group was demonstrated (Figure 7). The ratio of CD4/CD8 (Figure 7C) in mice treated with TEXomiR compared with the TEX- and PBS-treated mice was decreased by 0.53 and 0.41, respectively. This implies that TEXomiR induces more tumor-specific CTL response and decreases the ratio of CD4/CD8 in the tumor microenvironment. The evaluation of the CD4+CD25+Foxp3+ T cells determined that the administration of TEXomiR significantly (*p* < 0.05) decreased the tumor-infiltrated regulatory T cells compared with the PBS-treated group (Figure 8). There was a significant difference in the ratio of Treg/CD8 in the TEXomiR and TEX groups compared to the PBS group. The ratio of Treg/CD8 in mice treated with TEXomiR and TEX compared with the PBS-treated mice was decreased by 0.061 and 0.18, respectively. These data suggest that the subcutaneous injection of TEXomiR and TEX induces tumor-specific CTL response and reduces tumor-infiltrating Treg cells. Although TEX showed a significant effect in the reduction of tumor-infiltrated Treg cells, its efficacy and potency were less than those of TEXomiR. These findings highlight the synergism effect of TEX and miR-124 mimic in induction of anti-tumor immune responses.

In the field of tumor immunology, the CD4+CD25+ regulatory T cell expressing transcription factor Foxp3+ has been described to highly inhibit anti-tumor immunity (32). In murine mesotheliomas, a robust association between percentage of CD4+CD25+Foxp3+ T cells and tumor size has been demonstrated (32). Several studies have reported that either inhibiting a Treg-induced immunoregulatory pathway or depleting Treg cells might promote the efficacy of cancer immunotherapy (33, 34). *In vivo* depletion of regulatory T cells improved the effective anti-tumor immunity and accelerated the rejection of various immunogenic tumors in several strains of mice (34–36).

The survival analysis confirms that TEXomiR shows greater antitumor efficacy and consequently leads to improved survival of treated mice when compared with the TEX and PBS groups. Considerably, a prolonged survival rate with a long-lasting immune response was achieved in 100% of TEXomiR-treated mice at 46 days after the tumor challenge, whereas 33% of mice survived in the TEX-treated group, and no mice survived in the PBS treatment group, indicating that TEXomiRs are potent in inducing tumor-specific and long-lasting antitumor immunity.

A histopathological analysis of lymph nodes determined that, except for the TEXomiR group, initial tumor metastasis to the lymph nodes was identified in both the PBS and TEX groups. However, no considerable tumor metastasis to the lung was observed in any of the three groups. In most studies, in order to establish pulmonary and liver metastases, CT-26 cells have been injected in the tail vein of the mice (37, 38). Moreover, in one study for establishing metastatic liver tumors, CT-26 cells have been injected into the spleen (39). In our model, unlike the above-mentioned studies, the aim of the study was not solely to investigate the effect of TEXomiR on metastases. In our study, CT-26 tumor developed subcutaneously, and considering the type of treatment (miRNA based), the mice were sacrificed 30 days after tumor induction. It is possible that, at this time, metastases in the lung and liver are not enough. However, as shown in Figure 13, in the TEX and PBS groups, metastatic cells were identified in the lymph nodes and lymphatic vessels, which implies that these cells reach the bloodstream over time and then can migrate to the lung and liver.

The route of infusion and the milieu in which TEXs interact with immune cells are two key factors that determine the immune-inhibitory or immune-stimulatory effects of tumor exosomes. In the tumor microenvironment (TME), tumor cells in harmony with cancer-associated fibroblasts and stromal cells attenuate the induced antitumor immunity by production and recruitment of a wide range of soluble and insoluble immunosuppressive molecules (such as arginase I, IDO, ROS, IL-10, TGF-β,…) and cells (such as MDSCs and regulatory T cells). In this immunosuppressive setting, the secretion of tumor exosome synergizes with other immunosuppressive agents in TME, enhancing the progression and invasiveness of tumor or inhibiting the anti-tumor immune response. Hence, in the immunosuppressive conditions of TME, the interactions of TEXs and immune cells [T cells, APCs, natural killer (NK) cells…] not only have no significant effect on the induction of anti-tumor immune response but also suppress the effector functions of immune cells. The results of the MTT assay showed that treatment of tumor cells with TEXs in the absence of efficient immune effector cells promote the proliferation and invasiveness of these cells. In line with our results, several studies reported that tumor cell-derived exosomes increased tumor cell proliferation based on various *in vitro* assays (40–42). The study of Qu et al. reported that TEXs isolated from a gastric cancer cell line (SGC7901) significantly promoted the proliferation of two gastric cancer cell lines, BGC823 and SGC7901, based on MTT assay (40). In another investigation on gastric cancer cells, SGC-7901-derived exosomes enhanced SGC-7901 proliferation in a dose-dependent way (41).

As for their potential immunogenicity, the reasonable application of tumor-derived exosomes as a cell-free cancer vaccine has been proposed in several studies (43–45). Interestingly, Wolfers et al. reported that a wide spectrum of TEXs could be used as a source of tumor antigens for stimulating cytotoxic T lymphocytes and inducing an immune response more efficient than cell lysates in an animal model (44). This theory has also been approved in other tumor models including renal cell carcinoma, hepatocellular carcinoma (HCC), and leukemia (46–48). In line with our study, the study of Rao et al. indicated that DCs pulsed with HCC TEX could elicit a significantly robust immune response and inhibit tumor progression in HCC mice compared with cell lysate-pulsed DC (46). Based on the *in vitro* experiments, it has been shown that tumor exosomes may require the host DCs as an adjuvant for induction of potent immune responses (49–51). Hao et al. reported that the tumor cell or dendritic cell-derived exosomes' capability of stimulating efficient T-cell responses is completely missed in diphtheria toxin (DT)-treated DT receptor transgenic mice with the host dendritic cell deficiency (52), highlighting that tumor exosomes need the host DCs for transfer of their stimulatory effect to CD8 CTL responses *in vivo*. Selection of the source of antigens is important for tumor immunotherapy as no single antigen is ubiquitously expressed by CRC cells. There is a risk of immune evasion with a single peptide, and previous data with HLA-restricted antigens failed to trigger an antitumor immune response (53, 54). In contrast to single peptides, TEXs act as a source of multiple antigens for tumor immunotherapy. Consistent with these studies, our results demonstrated that, compared with the tumor lysate, TEX more efficiently induces the production of immunostimulatory cytokines and alters the immune milieu from immunoinhibitory to immunostimulatory (Figure 12). This is mainly due to a more complete and enriched collection of tumor antigens being presented in TEX compared to tumor lysate and may contribute to stimulate a larger number of T cell clones. In addition, efficient capture of TEX, processing in the MHC molecules, and prolonged presentation are other advantages of TEX (47). All these features are important in cancer immunotherapy (47). These results suggest that the context in which the immune system encounters TEX is essential in determining immunosuppression *vs*. immune stimulation.

The results of the cytokine assay revealed that the amount of IL12p70 and IFN-γ significantly increased in the TEXomiR group compared to the TEX and PBS groups (Figure 11). On the other hand, the levels of IL-4 and IL-10 significantly decreased in the TEXomiR group compared to the TEX and PBS groups. These indicated that miRNA-124 was able to polarize naïve CD4+ T cells into T helper type 1 and shift the cytokine balance from immunosuppressive to immune-stimulatory. An increased level of IL12p70 and IFN-γ, decreased levels of immunosuppressive IL-10 and IL-4, and elevated T-lymphocyte infiltration in tumor tissues in TEXomiR-treated mice do suggest that TEXomiR treatment elicits strong immunomodulatory effects. Our results demonstrated that TEXomiR, compared with TEX, could activate more functional T cells and elicit an adequate antitumor immune response to modulate the progression of CT-26-induced tumor.

In line with our results, it has been reported that miR-124 is an essential mediator for the polarization of CD4+ T cells into Th1 and Th17 cells *in vitro* and *in vivo*, which is controlled by the SOCS5 and upstream methyl CpG binding protein 2 (MeCP2) protein. Wei et al. reported that treatment of T cells with miR-124 mimic stimulates marked effector immune response including the overexpression of IFN-γ, TNF-α, IL-2, and STAT-3 (as a direct target of miR-124), which mediate immunosuppression in the tumor microenvironment (16). Actually, when miR-124 is overexpressed, Foxp3+ Treg and IL-17A + Th17 cell induction is suppressed, whereas differentiation of IFN-γ + Th1 cells is promoted (16). MiR-124, as a major tumor-suppressive miRNA, administers regulatory effects on several oncogenes and signaling pathways that are strictly related with tumor growth (55, 56). miR-124 applies tumor-suppressive roles on cell proliferation in non-small cell lung cancer (57), bladder cancer (58), and breast cancer (59). Furthermore, Liu et al. previously reported that miR-124 inhibits the growth and progression of CRC tumor through the direct targeting of inhibitor of apoptosis stimulatory protein phosphatase (11).

Our data also indicated a significant reduction of miR-124 target genes, STAT-3, SP1, and SOCS5, mRNA expression in splenocytes of mice treated with TEXomiR compared to the TEX and PBS groups. Considering this, miR-142-3p mimic led to a significant inhibition of STAT3 and SOCS5 in tumor tissues. Jiang *et al*. indicated that MeCP2 was essential for the naïve CD4+ T cells' differentiation into Th1 and Th17 subtests and also for Th1- or Th17-associated disorders *in vitro* and *in vivo* (14). Defective expression of MeCP2 in CD4+ T cells impaired the miR-124 expression and subsequently counteracted miR-124-mediated inhibition of the SOCS5 mRNA (14). Consequently, the accumulation of SOCS5 protein led to suppression of STAT-1 and STAT-3, which are indispensable for the differentiation of Th1 and Th17 cells, respectively (14). These data suggest that miR-124 acts as a potential factor in directing the anti-tumor immune responses. It has been shown that STAT-3 is constitutively expressed in various immune cells in the tumor microenvironment, and knocking out STAT-3 in tumor-infiltrating immune cells generates an intrinsic immune-surveillance system that prevents cancer cell proliferation and metastasis (60). In the study of Kortylewski et al. on tumor-bearing mice with STAT-3–/– hematopoietic cells, a significantly increased function of T cells, dendritic cells, neutrophils, and NK cells was represented (60). Accordingly, ablating Stat3 with small-molecule inhibitors induces NK cell- and T cell-dependent tumor growth inhibition (60).

Despite several strengths associated with this study, some limitations of the current study should be considered: firstly, due to the presence of exosomes in FBS and inaccessibility to exosome-free FBS, for resolving their interference with tumor exosomes, the CT-26 cells were adapted to FBS-free medium. Although the adaptation process did not considerably alter the phenotype and morphology of CT-26 cells, it may affect the quantity and quality of exosomes produced. Secondly, in this study, the transfection method was used to deliver miR-124-3p mimic into the exosome, and the cloning method was not applied to express miR-124 in the exosome. After we failed to get a successful loading efficiency by electroporation, we used a modified calcium chloride method for miRNA delivery, and its loading efficiency was moderate. Thirdly, although TEXomiR and TEX showed a significant tumor growth inhibition, no complete tumor eradication was identified in our study. Fourthly, our goal from the subcutaneous injection of TEXomiR and TEX was primarily to boost the DC-mediated immune response and subsequently promote the anti-tumor immune response. However, the exosomes produced had no specific targeting to DC and were not ligand-decorated exosomes, which may decrease the efficiency of treatment. Finally, the source and limited efficiency of exosome production, lack of targeting, and the miRNA loading process still remain unaddressed for possible clinical application of these interesting nanoparticles.

## Conclusion

Taken together, in this study, we used CT-26- derived exosomes as a source of multiple antigens for eliciting antitumor immune response and as natural carriers of miR-124-3p mimic delivery. It was identified that TEXomiR elicits potent antitumor immune response, which results in decreased tumor growth and prolonged survival rate. Applying TEXomiR, on one hand, took advantage of the unique feature of tumor exosomes carrying a broad spectrum of tumor antigens that could stimulate antigen-specific immune responses from both Th1 (CD4+ T cells) and CD8 cytotoxic T cells (CD8+ T cells), thus leading to an amplified immunotherapeutic effect of TEXomiR. On the other hand, miR-124 mimic, as a major tumor-suppressive miRNA, administers regulatory effects on several oncogenes and signaling pathways that are strictly related with tumor growth and promotes a T cell-dependent immune response.

## Data Availability Statement

The raw data supporting the conclusions of this article will be made available by the authors, without undue reservation.

## Ethics Statement

The animal study was reviewed and approved by this study was reviewed and approved by Ethical Committee and Research Advisory Committee of Shahid beheshti University of Medical Sciences (IR.SBMU.MSP.REC.1397.741).

## Author Contributions

RR contributed to execution, analysis, and interpretation of data. KB contributed to execution and participated in study design. SH participated in study design. MZ participated as clinical consultant. HG contributed to execution. DA participated in study design, manuscript drafting, and critical discussion. All authors contributed to the article and approved the submitted version.

## Conflict of Interest

The authors declare that the research was conducted in the absence of any commercial or financial relationships that could be construed as a potential conflict of interest.

## Supplementary Material

Supplementary Figure 1 Evaluation of tumor apoptosis in CT-26 tumor-bearing mice in the TEXomiR, TEX, and phosphate-buffered saline groups. The blue arrows indicate tumor apoptosis.
