# Supplementary figures and images for: Tumor-Derived Exosomes Enriched by miRNA-124 Promote Anti-tumor Immune Response in CT-26 Tumor-Bearing Mice
Source: Front Med (Lausanne). 2021 Apr 27;8:619939. doi: 10.3389/fmed.2021.619939 (PMC8110712; doi:10.3389/fmed.2021.619939)

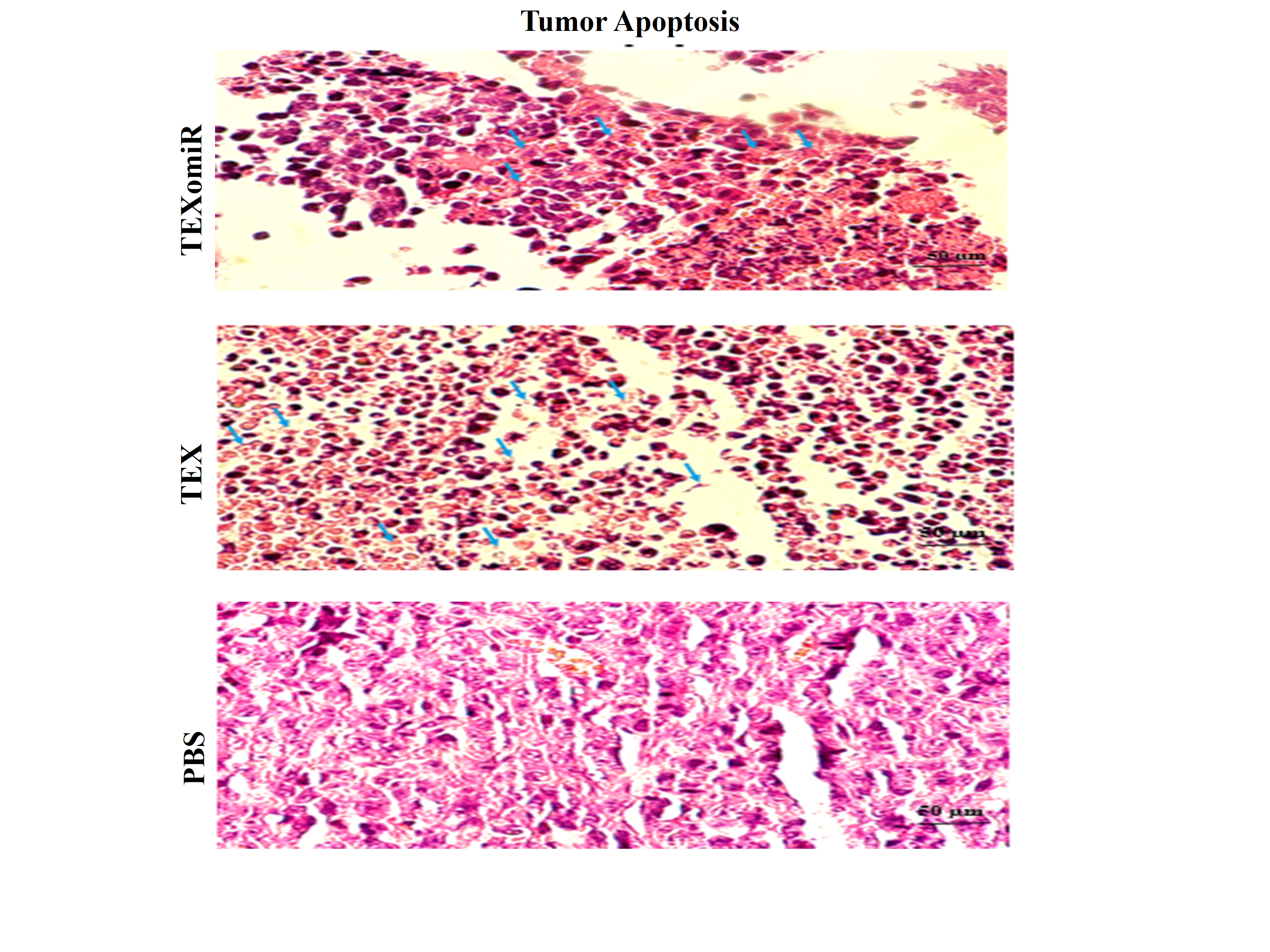

Supplement: Supplementary Figure 1 — Evaluation of tumor apoptosis in CT-26 tumor-bearing mice in the TEXomiR, TEX, and phosphate-buffered saline groups. The blue arrows indicate tumor apoptosis. [file Image_1.TIF]
